# Orientin Mitigates High Glucose/Ox‐LDL–Triggered Endothelial Cell Injury and Atherosclerosis by Regulating MARCH8‐Mediated NLRP3 Inflammasome Activation

**DOI:** 10.1155/mi/1841497

**Published:** 2026-03-27

**Authors:** Qi Li, Min Gao, Ni Zhong, Liang-Rong Zhang, Mei-Dong Zhu, Wei-Jing Ge, Qian-Zhu Wang, Xin Chen, Lei Zhang, Fu-Chen Song, Han-Zhi Lu

**Affiliations:** ^1^ Department of Vascular Surgery, Yueyang Hospital of Integrated Traditional Chinese and Western Medicine, Shanghai University of Traditional Chinese Medicine, Shanghai, China, shutcm.edu.cn; ^2^ Yichuan Community Health Service Center, Putuo District, Shanghai, China; ^3^ Department of Dermatology, Yueyang Hospital of Integrated Traditional Chinese and Western Medicine, Shanghai University of Traditional Chinese Medicine, Shanghai, China, shutcm.edu.cn

**Keywords:** atherosclerosis, diabetes, endothelial cell, MARCH8, NLRP3, Orientin

## Abstract

Endothelial cells under oxidative stress and inflammation are vital contributors to the progression of atherosclerosis. Although Orientin possesses antioxidant and anti‐inflammatory activities, the effects of Orientin on oxidized low‐density lipoprotein and high glucose (ox‐LDL/HG)–triggered endothelial cell injury and diabetes‐accelerated atherosclerosis remain unclear. ApoE^−/−^ mice were administered streptozotocin (STZ), fed with high‐fat diet (HFD), and then treated with Orientin to test the efficacy of Orientin on ameliorating atherosclerosis through pathological and biochemical assays. Human aortic endothelial cells (HAECs) were stimulated by ox‐LDL/HG followed by Orientin treatment, and the effects of Orientin on regulating HAEC viability, oxidative stress, inflammation, and endothelial‐mesenchymal transition (EndMT) were assessed using cell counting kit‐8 (CCK‐8), fluorescein diacetate (FDA) staining, quantitative real‐time PCR, immunofluorescence (IF), and western blot assays. The results showed that Orientin treatment decreased atherosclerotic plaque burden, lipid lesion, and collagen content in aortic and femoral arteries in diabetic mice. Meanwhile, Orientin alleviated hypercholesterolemia, as evidenced by decreased levels of total cholesterol, LDL‐cholesterol, and triglyceride. In HAECs, Orientin treatment increased cell viability and decreased inflammation, oxidative stress, and EndMT induced by ox‐LDL/HG. Furthermore, Orientin significantly inhibited reactive oxygen species (ROS)–triggered NOD‐like receptor family pyrin domain containing 3 (NLRP3) inflammasome activation and pyroptosis, as suggested by cleavage of caspase‐1 and gasdermin‐D (GSDMD), generation of interleukin (IL)‐18 and IL‐1β, and lactate dehydrogenase (LDH) release. Mechanistically, Orientin increased E3 ubiquitin ligase membrane‐associated RING‐CH 8 (MARCH8) expression in HAECs and resulted in subsequent MARCH8‐mediated ubiquitination and proteasomal degradation of the NLRP3 protein. Taken together, these data demonstrate that Orientin, which alleviates HAEC inflammation and pyroptosis through regulating the MARCH8/NLRP3 axis, might be a potential candidate for treating diabetes‐accelerated atherosclerosis.

## 1. Introduction

Globally, cardiovascular disease is a predominant health concern, responsible for an estimated 31% of all mortalities, which equates to ~17 million individuals annually [1]. Atherosclerosis is the common pathological basis for different kinds of cardiovascular diseases [1]. It is a chronic and progressive disease of large and medium‐sized arteries, characterized by the buildup of plaques formed by the deposition of cholesterol, other lipids, calcium, and macrophages in the innermost layer of the arteries [2, 3]. Atherosclerosis is based on three cornerstones: lipid metabolism alteration, inflammation, and endothelial injury [4]. Endothelial cells form a semipermeable barrier along the inner walls of blood vessels [5]. When there is an endothelial injury, lipoprotein particles pass through the endothelium and induce inflammatory reactions in macrophages and the covering endothelial cells, contributing to the progression of atherosclerosis [6]. Given the crucial role of endothelial cells in the initiation and progression of atherosclerosis, protecting these cells from damage could be an effective approach for atherosclerosis therapy.

Emerging evidence has demonstrated the therapeutic potential of medicinal natural products in cardiovascular disease [7]. For instance, flavonoids exert a protective effect against atherosclerosis due to their ability to inhibit the oxidation of low‐density lipoproteins [8]. Orientin, a flavonoid derived from medicinal plants, exhibits a range of medicinal properties, such as antioxidant, anti‐inflammatory, and antinociceptive effects [9]. Studies have demonstrated the potential use of Orientin in treating atherosclerosis. Li et al. [10] demonstrated that Orientin mitigates oxidized low‐density lipoprotein (ox‐LDL)‐induced inflammation and oxidative stress and inhibits foam cell formation. The function of Orientin in endothelial cells has also been explored. Orientin suppresses adhesion of monocytes, hyperpermeability, and expression of cell adhesion molecules (CAMs) in endothelial cells [11, 12]. Therefore, Orientin represents a promising therapeutic strategy for atherosclerosis that warrants further investigation.

NOD‐like receptor family pyrin domain containing 3 (NLRP3) is a sensor within the innate immune system [13]. Upon detecting endogenous cellular damage, it recruits apoptosis‐associated speck‐like protein (ASC) and caspase‐1 to form the NLRP3 inflammasome complex. The complex then cleaves caspase‐1 into its mature form, which subsequently leads to the cleavage of interleukin (IL)‐1β, IL‐18, and gasdermin‐D (GSDMD), resulting in pyroptosis [13, 14]. Overactivation of the NLRP3 inflammasome results in chronic low‐level inflammation, which is a typical characteristic of atherosclerosis [15]. NLRP3 inflammasome inhibition, through mechanisms such as microRNA [16], natural products [17], and posttranslational modification (PTM: ubiquitination, phosphorylation, etc.) [18], is an effective therapeutic strategy for atherosclerosis. Wang et al. [18] demonstrated that Oridonin inhibits atherosclerosis progression by preventing NFE2‐related factor 2 (Nrf2) ubiquitination and degradation and inhibiting NLRP3 inflammasome activation. Rho‐related GTP‐binding protein RhoE alleviates NLRP3‐dependent endothelial cell pyroptosis by regulating the ubiquitination and degradation of tumor necrosis factor receptor‐associated factor 6 (TRAF6) [19].

A recent study has revealed the roles of Orientin in increasing Nrf2 expression and inhibiting NLRP3 inflammasome activation in an acute lung injury model [20]. However, the regulatory role of Orientin in the NLRP3 inflammasome in atherosclerosis remains unclear. Based on the above findings, we aimed to investigate whether Orientin alleviates endothelial cell injury in atherosclerosis by regulating the NLRP3 inflammasome.

## 2. Materials and Methods

### 2.1. Animals

The work complied with the Animal Ethics Committee of the Shanghai University of Traditional Chinese Medicine (No. YYLAC‐2023‐355‐1) and was performed in compliance with the ARRIVE guidelines to minimize animal suffering. Male ApoE^−/−^ mice (C57BL/6 background, 8 weeks old) and C57BL/6 mice (8 weeks old) were obtained from Charles River Laboratories (Shanghai, China) and maintained in a pathogen‐free facility at 22 ± 2°C and 50 ± 10% relative humidity under a 12 h light–dark cycle with free access to water and food. ApoE^−/−^ mice were intraperitoneally (i.p.) administered streptozotocin (STZ) (50 mg/kg) for 5 consecutive days to induce diabetes. The blood glucose levels more than 16.7 mM were identified as diabetic 14 days after STZ injection [21]. Then, the diabetic mice were administered with high‐fat diet (HFD, D12492, Research Diets, NJ, USA). Control mice (C57BL/6, *n* = 5) were i.p. administered vehicle (citric acid buffer) and then administered with chow diet (D12450B, Research Diets). To assess the effects of Orientin on atherosclerosis, the diabetic mice were i.p. administered Orientin (50 mg/kg, *n* = 5) once every 2 days for 4 consecutive weeks in accordance with our preliminary experiments and previous studies [20, 21]. At the experimental endpoint, mice were euthanized by pentobarbital overdose followed by cervical dislocation, and blood, heart, aortas, and femoral arteries were harvested to carry out subsequent analysis.

### 2.2. Oil Red O Staining

To assess the atherosclerotic lesion development, the whole aortic tree was isolated, longitudinally dissected, and dyed with Oil Red O staining (Merck, MA, USA) according to the manufacturer’s instructions. Besides, femoral arteries were isolated and fixed in 4% PFA (Beyotime, Shanghai, China) overnight. Tissue sections (5 μm thick) were cut and then dyed with Oil Red O staining. Stained areas were quantitated with ImageJ software (NIH, MD, USA).

### 2.3. Hematoxylin‐Eosin (H&E) and Masson’s Trichrome Staining

Aortic sinus and femoral arteries were immobilized in 4% PFA, embedded in paraffin, sectioned at 5 μm thick, and then dyed with HE and Masson according to the suggestions provided for the Beyotime kit. Images were acquired using a DM500 optical microscope (Leica, Wetzlar, Germany). Plaque area from H&E, lipid area from Oil Red O, and collagen content from Masson were quantitated by Matlab (MathWorks, MA, USA).

### 2.4. Serum Biochemical Indices

Peripheral blood was collected from atherosclerotic and control mice. Total cholesterol (ab285242, Abcam, CA, USA), low‐density lipoprotein cholesterol (LDLc, Beyotime), and triglycerides (ab65336, Abcam) were assayed using the commercial kits listed above.

### 2.5. Overexpression and RNA Interference (RNAi) of Membrane‐Associated RING‐CH 8 (MARCH8)

The recombinant plasmids encoding full‐length human MARCH8 (pcDNA‐MARCH8) were obtained from Sangon Biotech (Shanghai, China) to overexpress MARCH8. pcDNA was used as a negative control. The plasmids were transfected into human aortic endothelial cells (HAECs) using Lipofectamine 3000 (Invitrogen, CA, USA) and validated. Small interfering RNA (siRNA) against MARCH8 (si‐MARCH8, CTTGAGCTGAATGAGAGAATA) was obtained from Genepharma (Shanghai, China) and transfected into HAECs using Lipofectamine RNAiMAX (Invitrogen) in accordance with the manufacturer’s protocol.

### 2.6. Cell Viability

HAECs were purchased from ScienCell (Carlsbad, CA, USA) and cultured in endothelial cell medium (ScienCell) containing 10% FBS (TianHang, Hangzhou, China). The cells were grown in a humidified CO2 incubator at 37°C. Cell viability was assessed using cell counting kit‐8 (CCK‐8) reagent (Beyotime) and fluorescein diacetate (FDA) staining (YEASEN, Shanghai, China), respectively. For the CCK‐8 assay, HAECs were seeded in 96‐well plates (10,000 cells/well) overnight and then treated with Orientin (0, 5, 10, 20, or 40 µM) alone or combined with ox‐LDL (50 mg/L, Mlbio, Shanghai, China) plus high glucose (HG, 30 mM of D‐glucose, Merck). After incubation for 24 h, cells were incubated with CCK‐8 (10 μL) for 2 h, and the absorbance at 450 nm was read with a ST‐360 microplate reader (KHB, Shanghai, China). For FDA staining, HAECs were seeded in 96‐well plates (5000 cells/well) overnight and then treated with Orientin (20 µM) in the presence or absence of ox‐LDL (50 mg/L, Mlbio) plus HG (30 mM of D‐glucose, Merck). After incubation for 24 h, cells were immobilized with 4% PFA for 10 min and incubated with FDA solution for 25 min. FDA‐positive cells were observed with a DMI4000B fluorescence microscopy (Leica).

### 2.7. Terminal Deoxynucleotidyl Transferase (TdT) dUTP Nick‐End Labeling (TUNEL)

To assess cell apoptosis, HAECs were seeded in 96‐well plates (10,000 cells/well) overnight and then treated with Orientin (20 µM) in the presence or absence of ox‐LDL (50 mg/L, Mlbio) plus HG (30 mM of D‐glucose, Merck). After treatment for 24 h, cells were immobilized with 4% PFA for 10 min and stained with TUNEL solution (YEASEN). TUNEL‐positive cells were observed with a DMI4000B fluorescence microscopy (Leica).

### 2.8. qRT‐PCR

After treatment with the specified reagents, HAECs were lysed with Trizol (Invitrogen) to collect total RNA. The first‐strand cDNA was synthesized in a 20 µL reaction system containing total RNA (2 µg), M‐MLV reverse transcriptase (Invitrogen), and oligo (dT) (TaKaRa). The protocol for cDNA synthesis was 10 min at 70°C, 2 min in ice water, and 65 min at 42°C. qRT‐PCR was performed on a 7500 real‐time PCR system (Applied Biosystems, CA, USA) in 20 µL reaction system containing cDNA, primers (Supporting Information 1: Table S1), and SYBR Green Supermix (Invitrogen). The protocol for qRT‐PCR was 5 min at 95°C, followed by 35 cycles of 15 s at 95°C and 15 s at 60°C. The mRNA levels were calculated by the 2^−ΔΔCT^ method [22] after normalization to β‐actin.

### 2.9. Western Blot

After treatment with the specified reagents, HAECs were lysed with RIPA buffer (Beyotime) to collect total protein. Western blot analysis was carried out in accordance with our previously described protocol [23]. The primary antibodies against CD31 (ab76533, Abcam), α‐SMA (PA5‐85070, Thermo Fisher Scientific), caspase‐1 (ab207802, Abcam), cleaved caspase 1 (PA5‐77886, Thermo Fisher Scientific), NLRP3 (ab263899, Abcam), ASC (ab309497, Abcam), GSDMD (ab210070, Abcam), GSDMD‐N (ab215203, Abcam), MARCH8 (PA5‐20632, Thermo Fisher Scientific), ubiquitin (ab179434, Abcam), and β‐actin (PA5‐78715, Thermo Fisher Scientific) were used in the study. The HRP‐coupled goat antirabbit IgG (H + L) (AS014, ABclonal, Wuhan, China) was used as the second antibody.

### 2.10. Glutathione (GSH) and Malonaldehyde (MDA) Measurements

HAECs were treated with the specified reagents, and the contents of GSH (ab239727) and MDA (ab118970) were measured by commercial ELISA kits according to the manufacturer’s instructions.

### 2.11. Immunofluorescence (IF)

HAECs were immobilized with 4% PFA on glass slides and treated with 0.1% Triton X‐100 (Beyotime) for 20 min. After incubation with 10% goat serum for 60 min and washing thrice with PBS, cells were treated with primary antibodies against CD31 (ab76533, Abcam) at 4°C overnight and then incubated with Alexa Fluor 488‐coupled secondary antibody for 1 h and DAPI (Merck) for 5 min in the dark. The fluorescence signals were observed with a DMI4000B fluorescence microscopy (Leica).

### 2.12. Intracellular Reactive Oxygen Species (ROS) Assay

HAECs were treated with Orientin (20 µM) in the presence or absence of ox‐LDL (50 mg/L, Mlbio) plus HG (30 mM of D‐glucose, Merck). Twenty‐four hours later, cells were incubated with 20 μM of H2DCFDA (ab113851, Abcam) for 45 min at room temperature. Intracellular ROS was observed with a DMI4000B fluorescence microscopy (Leica).

### 2.13. Coimmunoprecipitation (Co‐IP)

HAECs were treated with NP40 lysis buffer (Invitrogen). The cell lysate was incubated with an anti‐NLRP3 antibody (ab263899, Abcam) or IgG control for 2 h and then incubated with protein A/G agarose beads (ab286842, Abcam) overnight. Beads were collected by centrifugation (12,000 rpm and 5 min) and washed three times using washing buffer. The immunocomplexes were eluted and then used as western blot assay with the MARCH8 antibody (PA5‐20632). The lysate (10%) was used as a positive control (input).

### 2.14. Cycloheximide (Chx) Chase Experiment

After transfection with 3 ng of pcDNA‐MARCH8 or pcDNA control for 24 h, HAECs were incubated with 100 μg/mL of Chx (MCE, NJ, USA). Subsequently, total protein was isolated after 0, 3, 6, or 9 h exposure to Chx, and the NLRP3 protein was assessed using western blot.

### 2.15. Statistics

Each experiment was conducted a minimum of three times, with the results presented as the mean ± standard deviation (SD). The Student’s *t*‐test and one‐way ANOVA (followed by Dunnett’s test) were employed to analyze differences between two groups and among three groups, respectively, using GraphPad Prism 7.0 (CA, USA). The threshold for significance was set at *p* < 0.05.

## 3. Results

### 3.1. Orientin Alleviated Atherosclerosis and Hypercholesterolemia in Diabetic Mice

To explore the biological function of Orientin in ameliorating atherosclerosis, diabetic mice were established and treated with Orientin. En face analysis of Oil Red O staining showed that Orientin treatment prominently alleviated atherosclerotic plaque burden along the aortic tree compared with vehicle control (Figure 1A,B). The role of Orientin in atherosclerosis development in the aortic sinus was evaluated through HE, Oil Red O, and Masson staining. The results from HE staining revealed that Orientin decreased plaque area (Figure 1C,D). Lipid lesions were significantly decreased by Orientin through Oil Red O staining (Figure 1E,F). Collagen content was significantly increased by Orientin through Masson staining (Figure 1C,D).

Figure 1Orientin decreased atherosclerotic plaque development in the aortas of diabetic mice. The aortic trees and aortic sinuses were obtained from control mice, diabetic mice treated with vehicle, and diabetic mice treated with Orientin (*n* = 5 per group). (A and B) Atherosclerotic plaque sizes were assessed in aortic trees by en face Oil Red O staining. (C and D) Plaque area in aortic sinuses was assessed by H&E staining and quantitated (normalized to total sinus area). (E and F) Lipid lesion area in aortic sinuses was assessed by Oil Red O staining and quantitated. (G and H) Collagen content in aortic sinuses was assessed by Masson staining and quantitated.  ^∗^ *p* < 0.05,  ^∗∗^ *p* < 0.01, and  ^∗∗∗^ *p* < 0.001.

**Figure:**
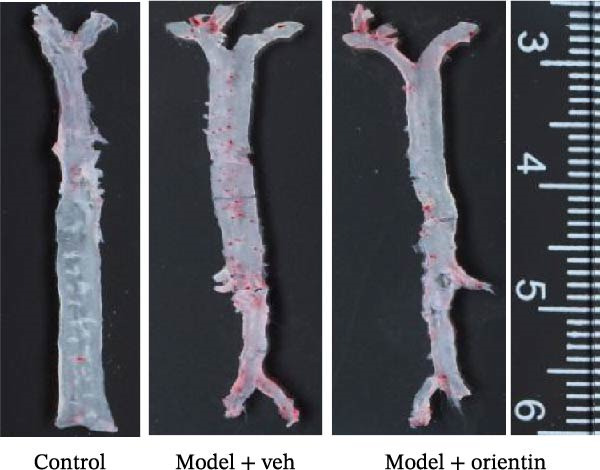
(A)

**Figure:**
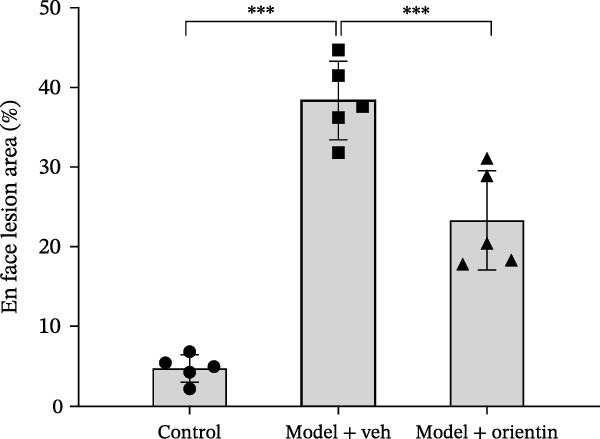
(B)

**Figure:**
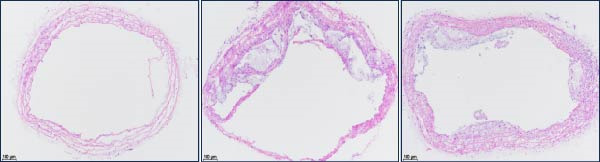
(C)

**Figure:**
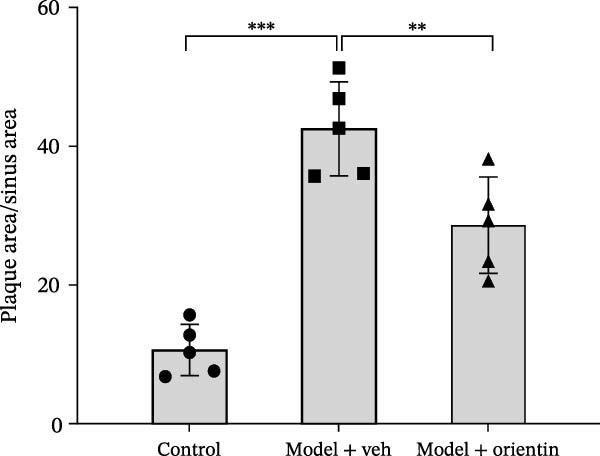
(D)

**Figure:**
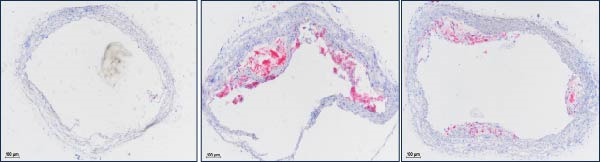
(E)

**Figure:**
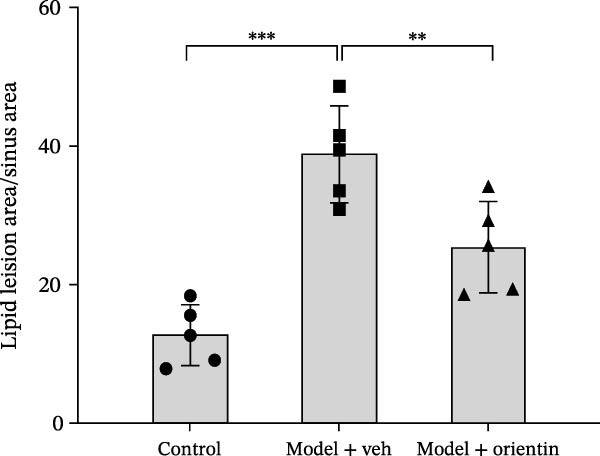
(F)

**Figure:**
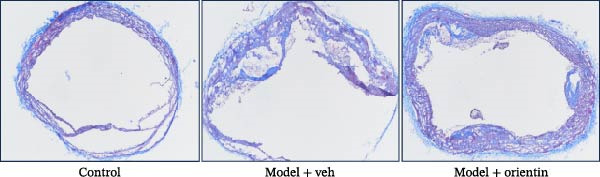
(G)

**Figure:**
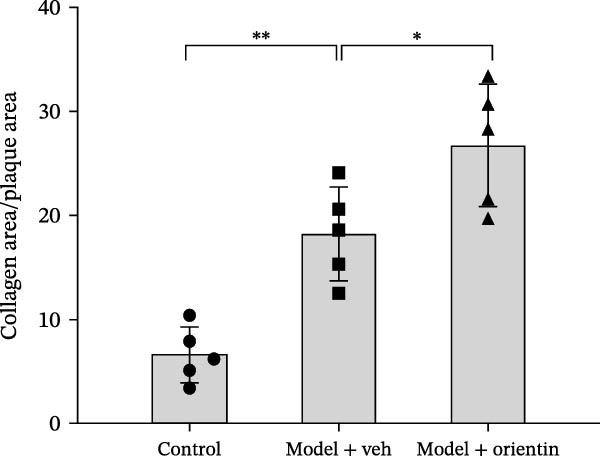
(H)

Besides, the effects of Orientin on ameliorating atherosclerosis in the femoral artery were further assessed. As shown in Figure 2A,B, lipid lesions in the femoral artery were significantly increased in diabetic mice, whereas Orientin ameliorated atherosclerotic injury in the femoral artery. Moreover, diabetic mice exhibited hypercholesterolemia as suggested by increased levels of total cholesterol, LDL‐cholesterol, and triglyceride, whereas the effects were reversed by Orientin (Figure 2C–E). These results demonstrate that Orientin treatment ameliorates atherosclerosis and hypercholesterolemia in diabetic mice.

Figure 2Orientin inhibited atherosclerotic plaque development in femoral arteries. The femoral arteries were obtained from control mice (*n* = 5) and diabetic mice treated with vehicle (*n* = 5) or Orientin (50 mg/kg, *n* = 5). (A and B) Lipid lesion area was assessed by Oil Red O staining and quantitated. Total cholesterol (C), LDLc (D), and triglycerides (E) were assessed by commercial kits.  ^∗^ *p* < 0.05,  ^∗∗^ *p* < 0.01, and  ^∗∗∗^ *p* < 0.001.

**Figure:**
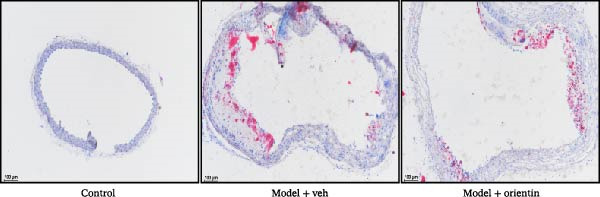
(A)

**Figure:**
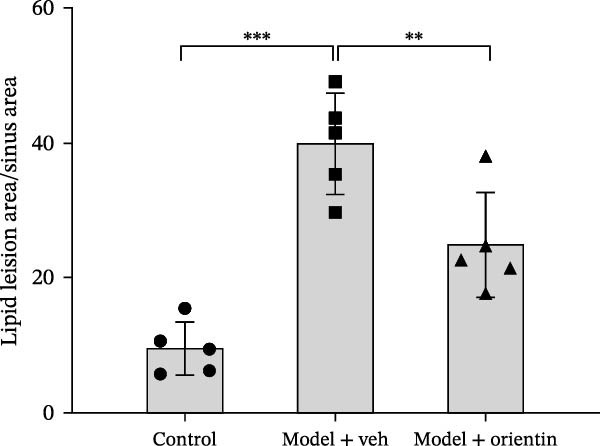
(B)

**Figure:**
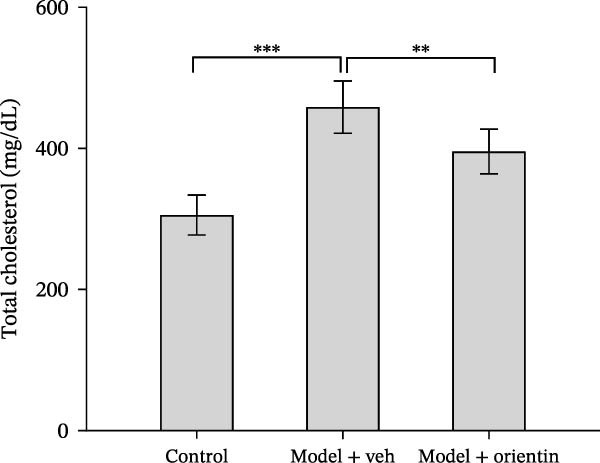
(C)

**Figure:**
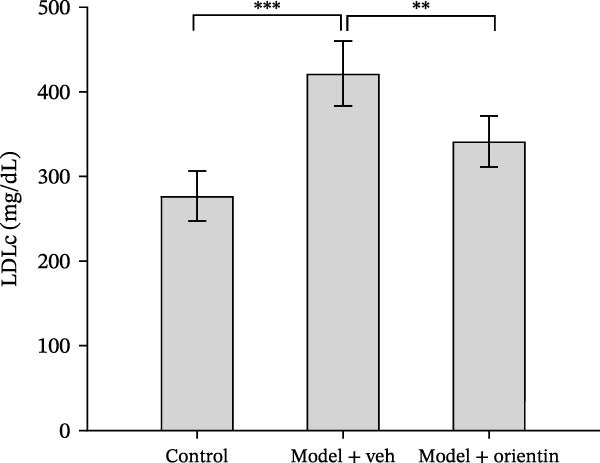
(D)

**Figure:**
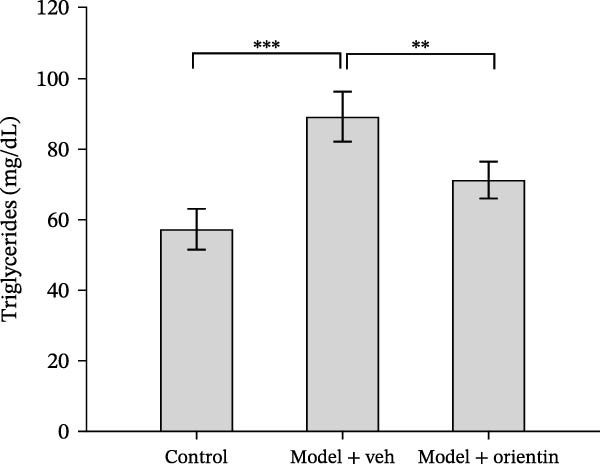
(E)

### 3.2. Orientin Decreased HAEC Apoptosis and Inflammation Following the Oxidized Low‐Density Lipoprotein and High Glucose (Ox‐LDL/HG) Challenge

Endothelial damage is a critical and early step in the development of diabetes‐accelerated atherosclerosis [24, 25]. The roles of Orientin in regulating HAEC apoptosis and inflammation after ox‐LDL/HG treatment were next investigated. The results from CCK‐8 assay showed that Orientin increased HAEC viability in a dose‐dependent manner in the presence of ox‐LDL/HG (Figure 3A). The results from FDA staining revealed that ox‐LDL/HG decreased HAEC viability (Figure 3B,C). Ox‐LDL/HG treatment resulted in significant HAEC apoptosis (Figure 3D,E). All these effects were blocked by Orientin (Figure 3B–E). Inflammation triggered by ox‐LDL/HG is an important factor in endothelial dysfunction [26]. As shown in Figure 3F–I, ox‐LDL/HG induced an obvious proinflammatory response in HAECs, as suggested by increased expression levels of IL‐6, tumor necrosis factor (TNF)‐α, intercellular adhesion molecule (ICAM)‐1, and vascular CAM (VCAM)‐1, whereas Orientin reversed the effects.

Figure 3Orientin decreased HAEC apoptosis and inflammation following the ox‐LDL/HG challenge. (A) HAECs were treated with ox‐LDL/HG (50 mg/L of ox‐LDL and 30 mM of HG) alone or combined with Orientin (0, 5, 10, 20, or 40 µM) for 24 h, and then cell viability was measured by CCK‐8. (B and C) HAECs were treated with ox‐LDL/HG alone or combined with Orientin (20 µM) for 24 h, and then cell viability was measured by FDA staining. (D and E) HAECs were treated with ox‐LDL/HG alone or combined with Orientin (20 µM) for 24 h, and then cell apoptosis was measured by TUNEL. HAECs were treated with ox‐LDL/HG alone or combined with Orientin (20 µM) for 24 h, and then the mRNA levels of IL‐6 (F), TNF‐α (G), ICAM‐1 (H), and VCAM‐1 (I) were measured by qRT‐PCR analysis.  ^∗^ *p* < 0.05, and  ^∗∗^ *p* < 0.01.

**Figure:**
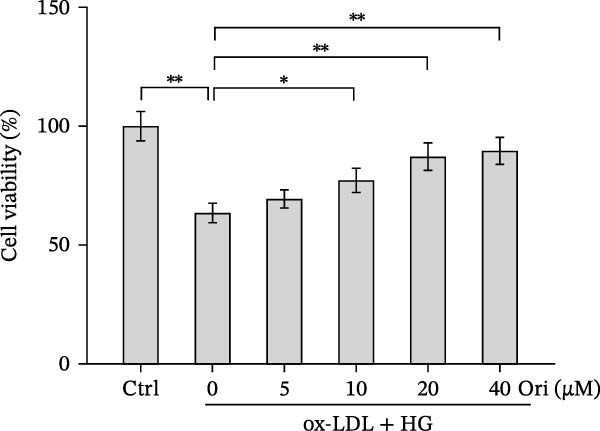
(A)

**Figure:**
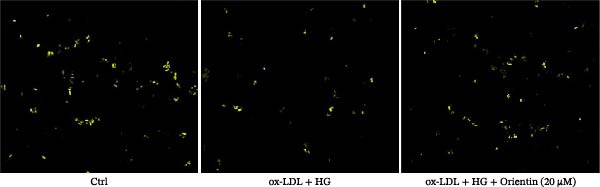
(B)

**Figure:**
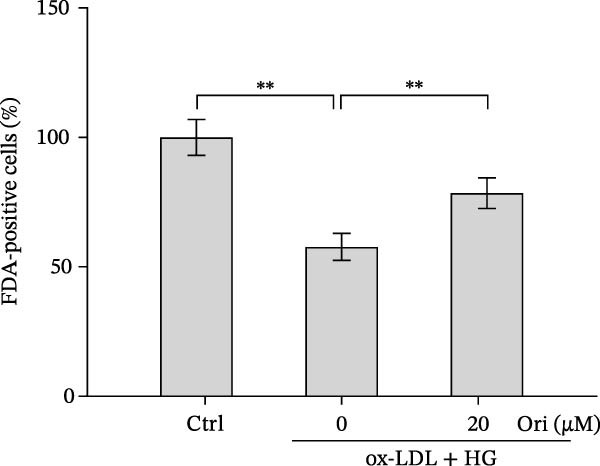
(C)

**Figure:**
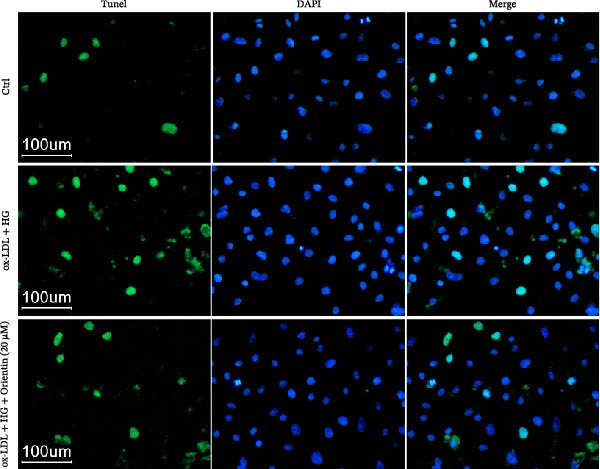
(D)

**Figure:**
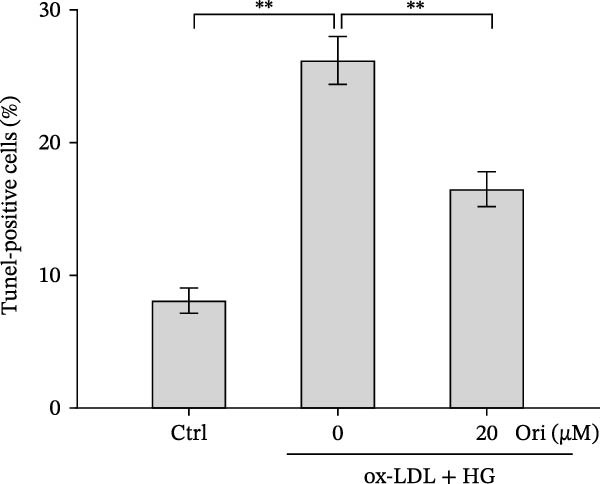
(E)

**Figure:**
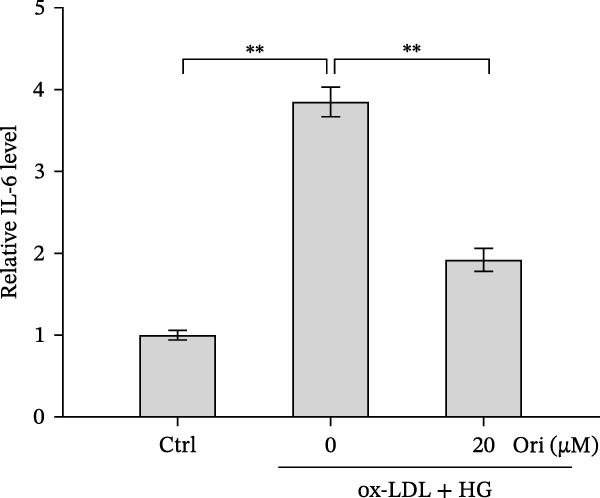
(F)

**Figure:**
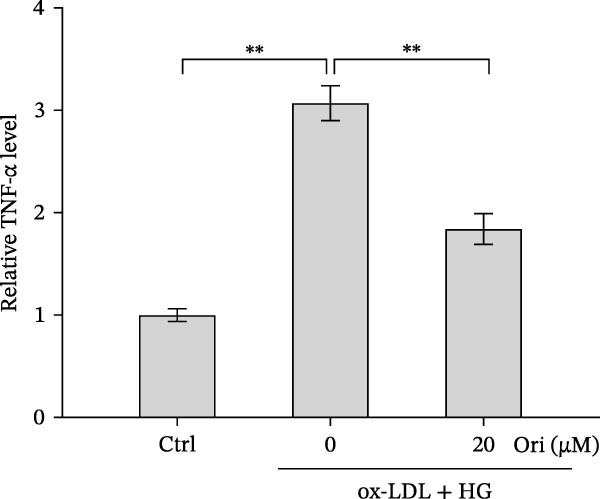
(G)

**Figure:**
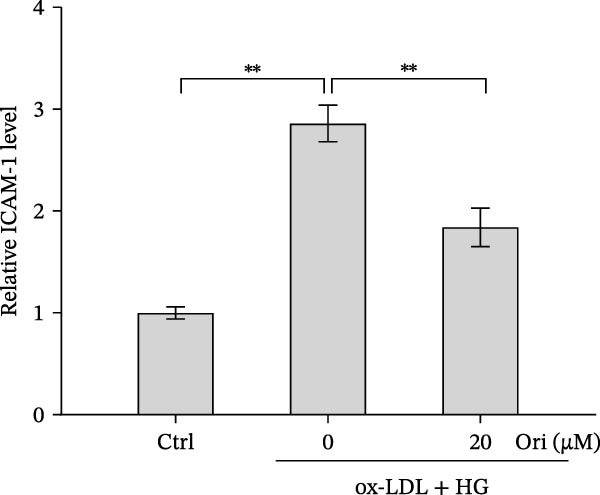
(H)

**Figure:**
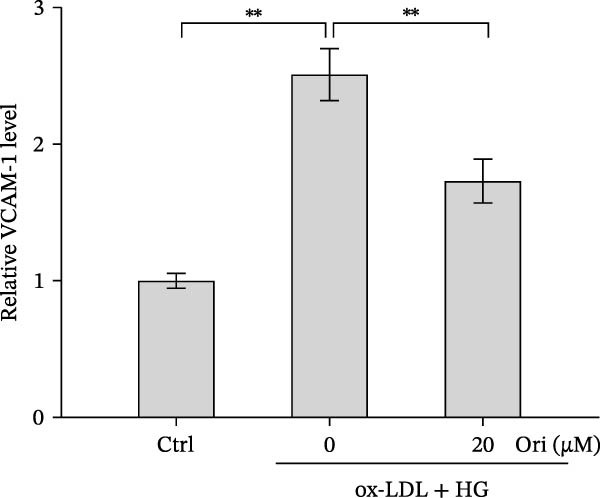
(I)

### 3.3. Orientin Protected Against Ox‐LDL/HG–Induced Oxidative Stress and Endothelial‐Mesenchymal Transition (EndMT)

Given the roles of oxidative stress [21, 27] and EndMT [28, 29] on endothelial dysfunction, we further assessed whether Orientin alleviated ox‐LDL/HG–induced oxidative stress and EndMT. Exposure to ox‐LDL/HG markedly increased ROS levels (Figure 4A,B), accelerated MDA production (a marker of oxidative stress, Figure 4C), and decreased GSH levels (Figure 4D) in HAECs. All these effects were reversed by Orientin (Figure 4A–D). Besides, exposure to ox‐LDL/HG resulted in a significant decrease in endothelial markers (CD31 and vWF) and an increase in mesenchymal markers (α‐SMA and vimentin) in HAECs at the mRNA levels, where these effects were blocked by Orientin (Figure 4E,F), suggesting that Orientin suppressed ox‐LDL/HG–induced EndMT. The results from western blot (Figure 4G,H) and IF (Figure 4I) analysis further showed that Orientin restored CD31 protein levels and inhibited α‐SMA protein levels in ox‐LDL/HG–challenged HAECs.

Figure 4Orientin protected against ox‐LDL/HG–induced oxidative stress and EndMT. HAECs were treated with ox‐LDL/HG alone or combined with Orientin (20 µM) for 24 h. (A and B) The ROS levels were measured using H2DCFDA fluorescent dye. The content of MDA (C) and GSH (D) was measured using commercial ELISA kits. (E and F) The mRNA levels of CD31, vWF, α‐SMA, and vimentin were measured using qRT‐PCR analysis. (G and H) The protein levels of CD31 and α‐SMA were measured using western blot analysis. (I) The protein expression of CD31 and α‐SMA was measured using IF analysis.  ^∗^ *p* < 0.05, and  ^∗∗^ *p* < 0.01.

**Figure:**
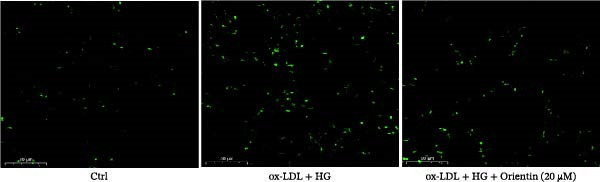
(A)

**Figure:**
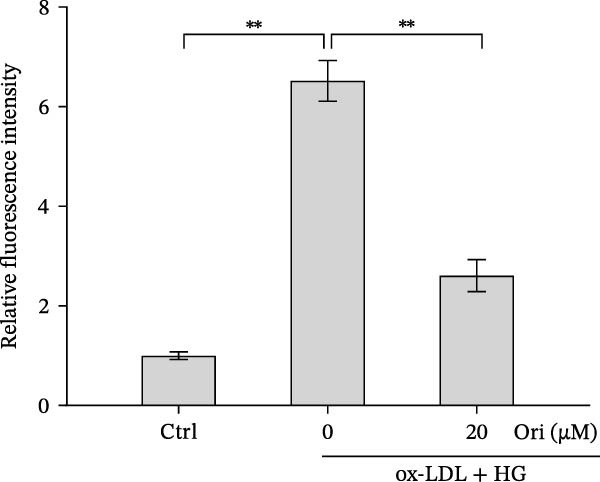
(B)

**Figure:**
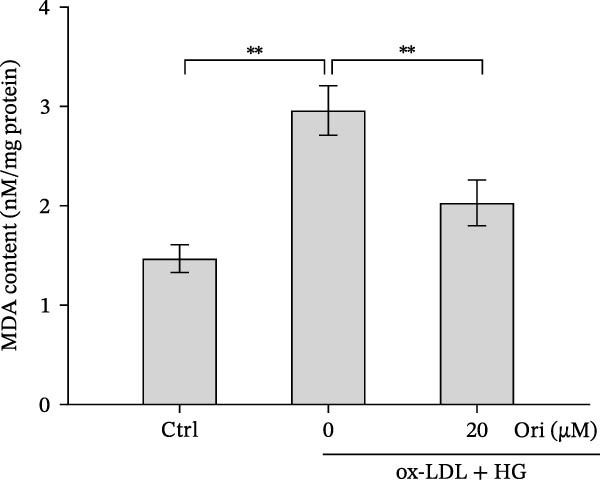
(C)

**Figure:**
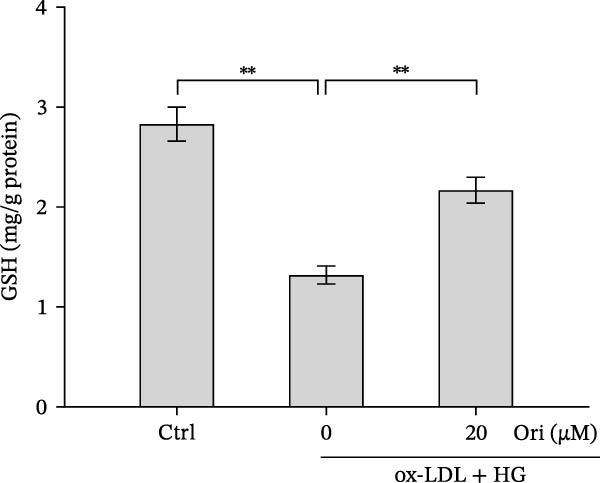
(D)

**Figure:**
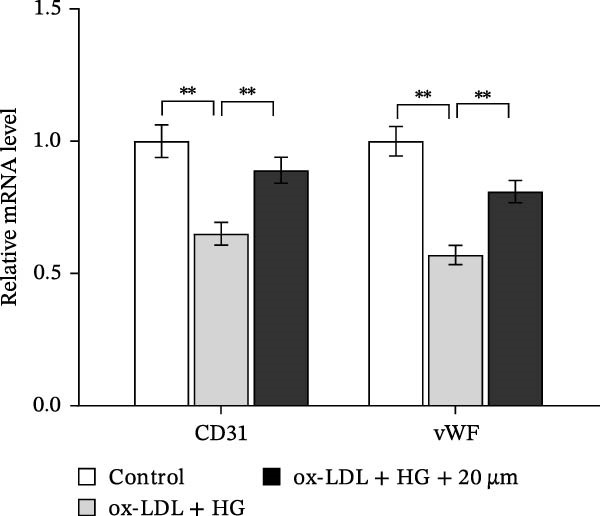
(E)

**Figure:**
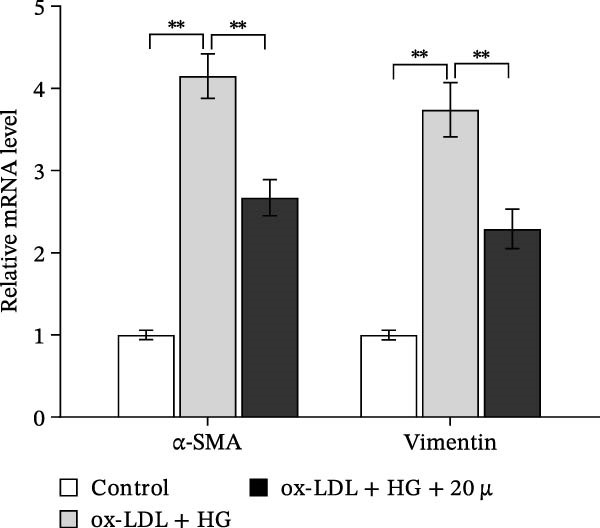
(F)

**Figure:**
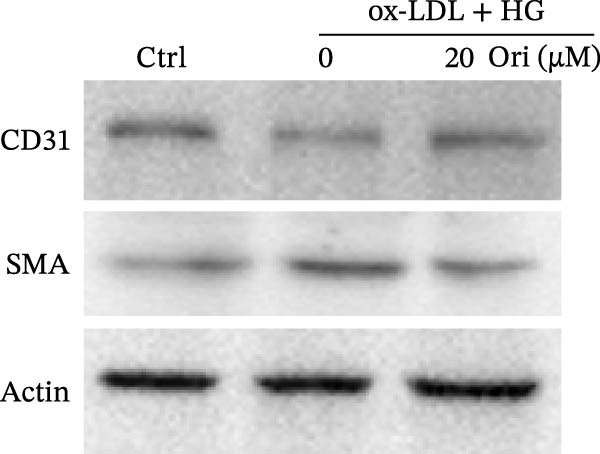
(G)

**Figure:**
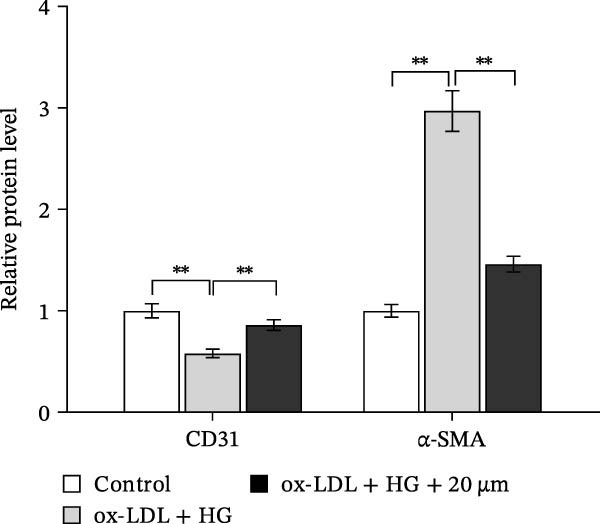
(H)

**Figure:**
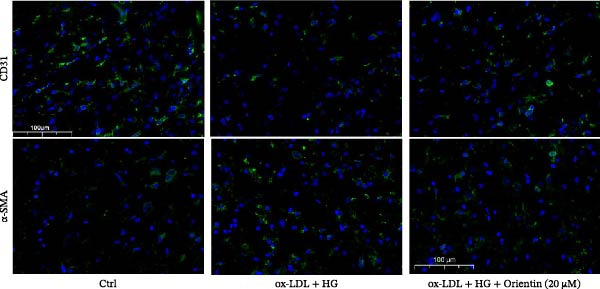
(I)

### 3.4. Orientin Attenuated ROS‐Triggered Pyroptosis in HAECs

ROS accelerates cell death through promoting apoptosis [30], ferroptosis [31], necroptosis [32], pyroptosis [33], and autophagy [34]. In HAECs, ox‐LDL/HG–induced cell death was blocked by the inhibitors of apoptosis (ZVAD‐FMK), necroptosis (Nec‐1), and pyroptosis (disulfiram) (Figure 5A). Given the dual effects of Orientin on regulating inflammation and cell viability, both of which are correlated with the inflammasome, the roles of Orientin in regulating ox‐LDL/HG–induced inflammasome activation were further investigated. Ox‐LDL/HG resulted in caspase‐1 cleavage, whereas these effects were blocked by NAC (a ROS scavenger) (Figure 5B), indicating NAC inhibited ox‐LDL/HG–induced inflammasome activation. As expected, Orientin also alleviated ox‐LDL/HG–triggered inflammasome activation, as evidenced by a decrease in caspase‐1 cleavage (Figure 5C,D) and IL‐18 and IL‐1β production (Figure 5E,F). Orientin further inhibited ox‐LDL/HG–triggered pyroptosis, as suggested by the decrease in lactate dehydrogenase (LDH) release (Figure 5G) and GSDMD cleavage (Figure 5H,I).

Figure 5Orientin attenuated ROS‐triggered pyroptosis in HAECs. (A) HAECs were treated with ox‐LDL/HG alone or combined with vehicle, ZVAD‐FMK (ZVAD, 10 μM), ferrostatin‐1 (Fer‐1, 1 μM), necrostatin‐1 (Nec‐1, 10 μM), disulfiram (1 μM), or 3‐MA (10 mM) for 24 h, and then cell viability was measured by CCK‐8. (B) HAECs were treated with ox‐LDL/HG alone or combined with NAC (5 mM), and then the protein levels of caspase‐1 and cleaved caspase‐1 were measured using western blot analysis. HAECs were treated with ox‐LDL/HG alone or combined with Orientin (20 µM). (C and D) The protein levels of caspase‐1 and cleaved caspase‐1 were measured using western blot analysis. (E and F) The mRNA levels of IL‐18 and IL‐1β were measured using qRT‐PCR analysis. (G) LDH production was assessed by ELISA analysis. (H and I) The protein levels of full‐length GSDMD (GSDMD‐F) and its N‐terminal domain (GSDMD‐N) were measured using western blot analysis.  ^∗^ *p* < 0.05, and  ^∗∗^ *p* < 0.01.

**Figure:**
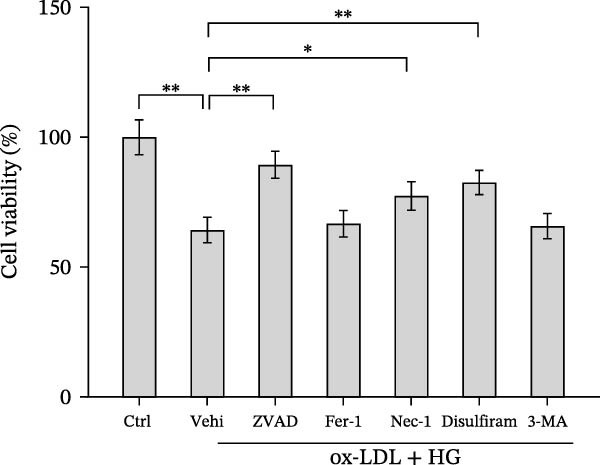
(A)

**Figure:**
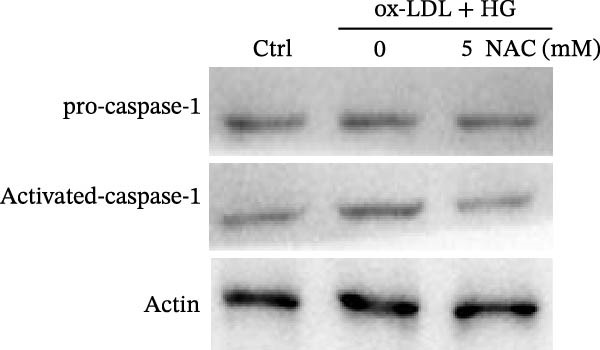
(B)

**Figure:**
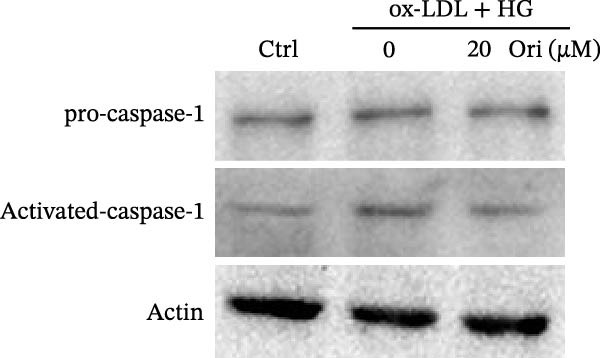
(C)

**Figure:**
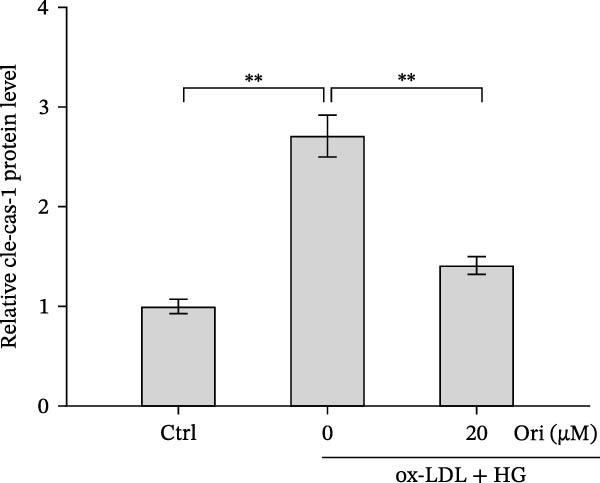
(D)

**Figure:**
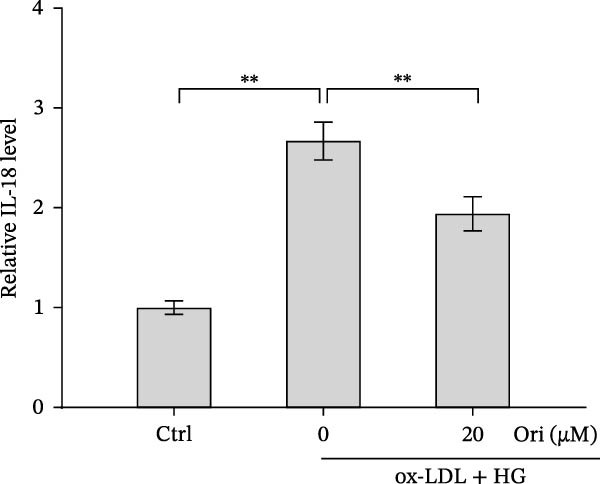
(E)

**Figure:**
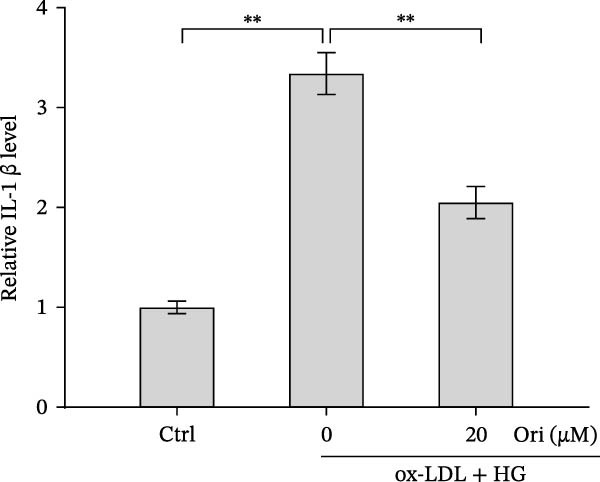
(F)

**Figure:**
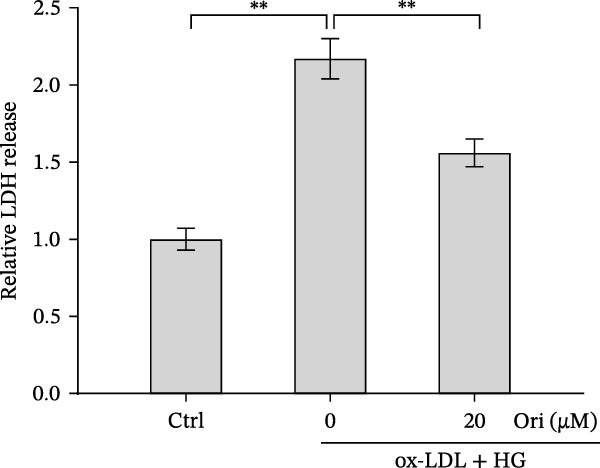
(G)

**Figure:**
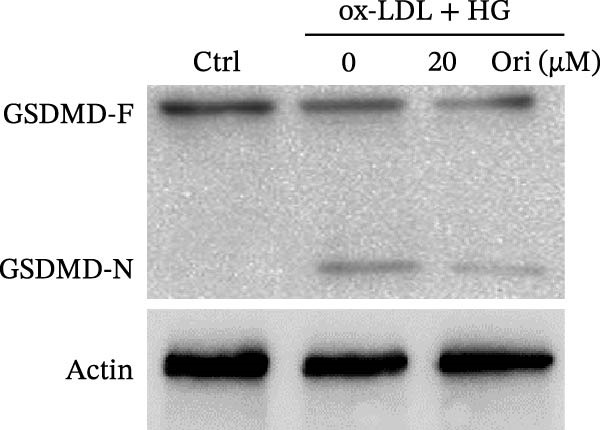
(H)

**Figure:**
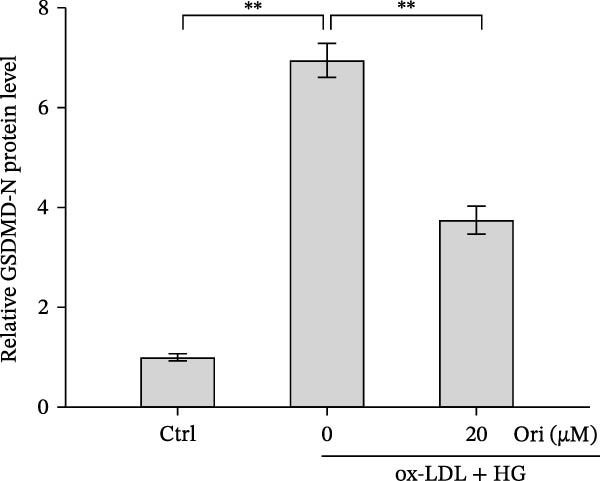
(I)

### 3.5. Orientin Inhibited NLRP3 Inflammasome Activation

To identify which inflammasome is suppressed by Orientin, the effects of Orientin on inflammasome‐mediated IL‐1β and IL‐18 production were assessed in HAECs following treatment with NLRP3 inflammasome agonists (alum and nigericin), AIM2 inflammasome agonists (poly(dA:dT)), NLRC4 inflammasome agonists (flagellin), or NLRP1 inflammasome agonists (MDP) [35, 36]. These inflammasome agonists all increased IL‐1β and IL‐18 production compared with control, and NLRP3 inflammasome agonists (alum and nigericin) exhibited the strongest effects (Figure 6A,B). More importantly, compared with vehicles, Orientin‐treated HAECs produced markedly lower levels of IL‐1β and IL‐18 in response to alum or nigericin (Figure 6A,B). Therefore, the regulatory effects of Orientin on NLRP3 were next investigated. Although Orientin did not affect the mRNA levels of NLRP3 and ASC in oxLDL/HG–treated HAECs (Figure 6C), the protein levels of NLRP3 were markedly inhibited by Orientin (Figure 6D,E).

Figure 6Orientin inhibited NLRP3 inflammasome activation. (A and B) HAECs were treated with ox‐LDL/HG alone or combined with alum (200 µg/mL), nigericin (2 µg/mL), poly(dA:dT) (5 µg/mL), flagellin (2 µg/mL), or muramyl dipeptide (MDP, 5 µg/mL) for 24 h, and then the mRNA levels of IL‐18 and IL‐1β were measured using qRT‐PCR analysis. HAECs were treated with ox‐LDL/HG alone or combined with Orientin (20 µM), and then the mRNA (C) and protein (D and E) levels of NLRP3 and ASC were assessed using qRT‐PCR and western blot analysis, respectively. HAECs were overexpressed with MARCH8, and then the mRNA (F) and protein (G) levels of NLRP3 were assessed using qRT‐PCR and western blot analysis, respectively. HAECs were treated with Orientin (20 µM) alone or combined with siRNAs against MARCH8 (siMARCH8, 50 nM), and then the mRNA (H) and protein (I) levels of NLRP3 were assessed using qRT‐PCR and western blot analysis, respectively.  ^∗^ *p* < 0.05,  ^∗∗^ *p* < 0.01, and  ^∗∗∗^ *p* < 0.001.

**Figure:**
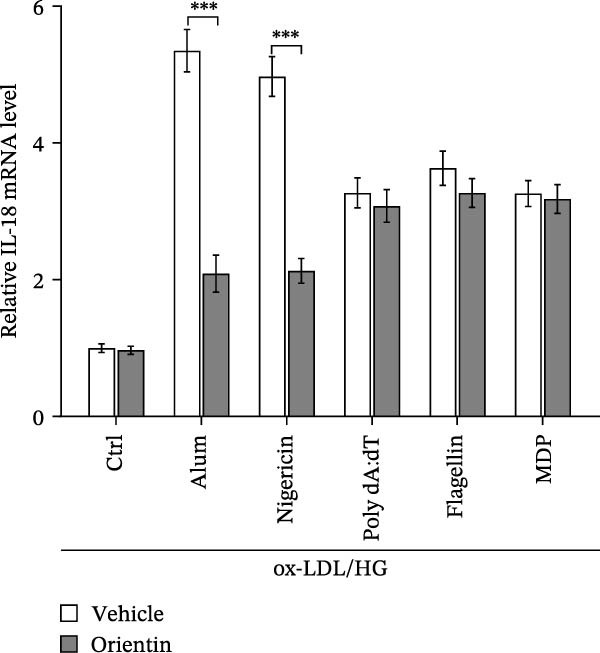
(A)

**Figure:**
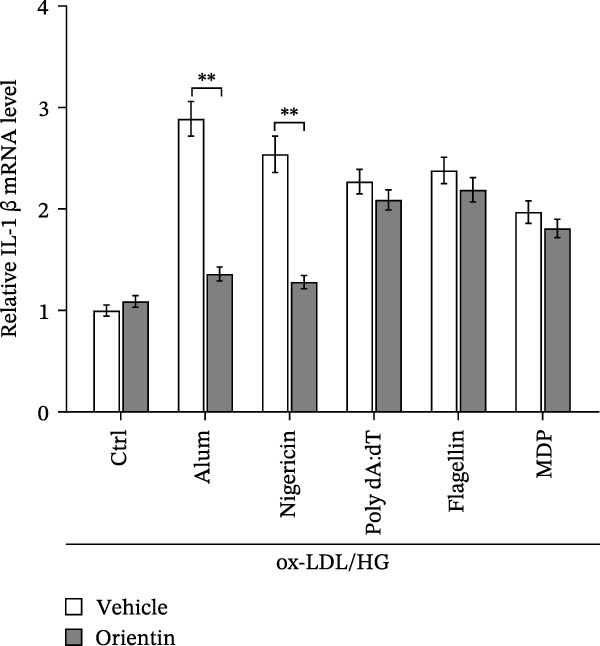
(B)

**Figure:**
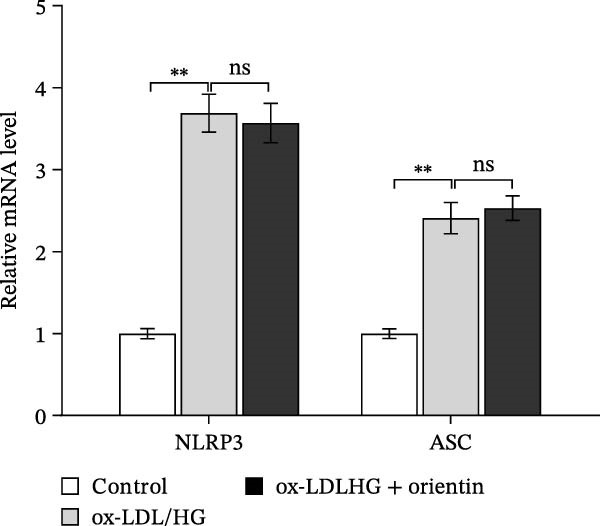
(C)

**Figure:**
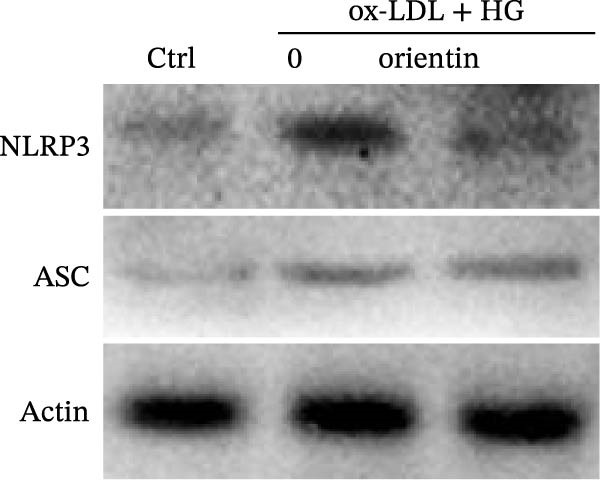
(D)

**Figure:**
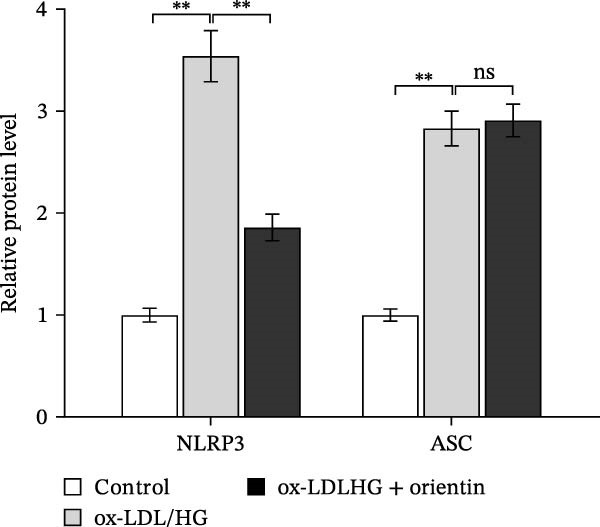
(E)

**Figure:**
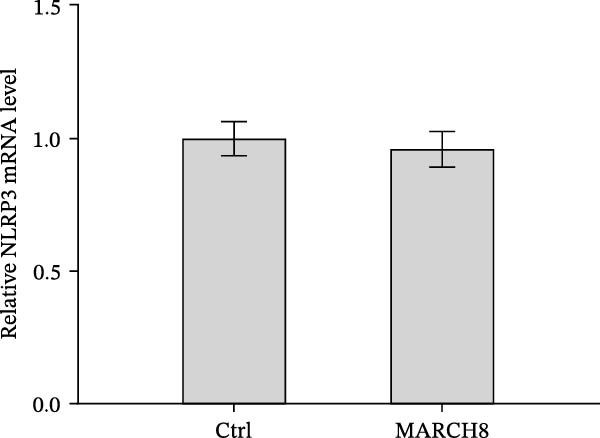
(F)

**Figure:**
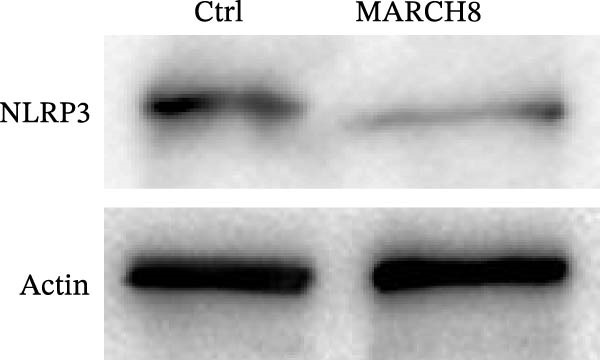
(G)

**Figure:**
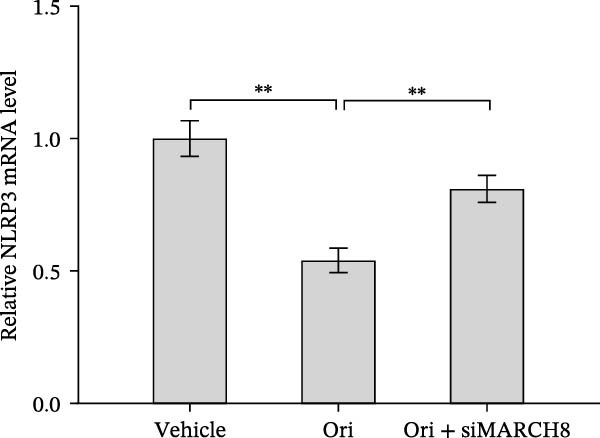
(H)

**Figure:**
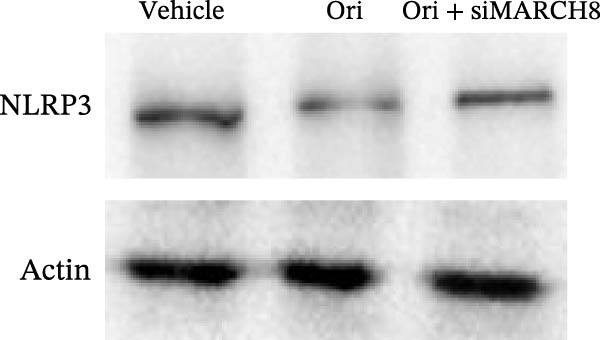
(I)

Ubiquitination is the best‐characterized mechanism of PTMs and plays a critical role in regulating NLRP3 inflammasome activation [37]. Forty‐one E3 ubiquitin ligases targeting the NLRP3 protein were predicted by the UbiBrowser tool (http://ubibrowser.bio-it.cn/ubibrowser/) (Supporting Information 2: Table S2). Differentially expressed genes were identified in oxLDL/HG–treated endothelial cells using a publicly available dataset (GSE173669) (Supporting Information 3: Table S3). Venn diagram analysis revealed that three E3 ubiquitin ligases (CBLC, CRYAB, and MARCH8) were dysregulated in the GSE173669 dataset (Supporting Information 4: Figure S1A). Orientin treatment increased MARCH8 expression at the mRNA levels (Supporting Information 4: Figure S1B) and protein levels (Supporting Information 4: Figure S1C) but did not affect CBLC and CRYAB expression. As expected, MARCH8 overexpression did not change NLRP3 mRNA levels (Supporting Information 5: Figure S2A,C, Figure 6F) but significantly inhibited NLRP3 protein expression (Figure 6G,H), indicating that MARCH8 might promote ubiquitination and proteasomal degradation of NLRP3 protein. Furthermore, Orientin treatment increased NLRP3 protein expression, whereas these effects were reversed by MARCH8 knockdown (Supporting Information 5: Figure S2B,C, Figure 6I). These results demonstrate that Orientin inhibits NLRP3 inflammasome activation by increasing MARCH8.

### 3.6. MARCH8 Promoted Ubiquitination and Proteasomal Degradation of the NLRP3 Protein

Finally, we investigated whether MARCH8 inhibited NLRP3 protein expression through accelerating ubiquitin‐mediated degradation. The Co‐IP assay was applied to assess the direct coupling of MARCH8 with NLRP3 in HAECs, and the results confirmed this assumption (Figure 7A). MARCH8 overexpression obviously promoted the ubiquitination of NLRP3 proteins in HAECs (Figure 7B) by IP assay with NLRP3 antibodies and subsequent western blot assay with ubiquitin antibodies. As a result, MARCH8 overexpression prominently decreased the half‐life of NLRP3 proteins and the NLRP3 proteins’ stability (Figure 7C,D). Furthermore, MG132, a proteasome inhibitor, alleviated MARCH8‐mediated degradation of NLRP3 proteins (Figure 7E). These results demonstrate that MARCH8 facilitates the ubiquitination and proteasomal degradation of NLRP3 protein in HAECs.

Figure 7MARCH8 promoted ubiquitination and proteasomal degradation of the NLRP3 protein. (A) HAEC extracts were subjected to a Co‐IP assay with an anti‐NLRP3 antibody, followed by a western blot assay with the anti‐MARCH8 antibody. (B) HAECs were transfected with pcDNA‐MARCH8 or pcDNA control. The extracts were subjected to a Co‐IP assay with the anti‐NLRP3 antibody, followed by a western blot assay with the antiubiquitin antibody. (C and D) After transfection with pcDNA‐MARCH8 or pcDNA control, HAECs were incubated with 100 μg/mL of Chx. Total protein was isolated after 0, 3, 6, or 9 h exposure to Chx, and the NLRP3 protein was assessed using western blot analysis. (E) HAECs were treated with pcDNA‐MARCH8 alone or combined with MG132 (20 µM), and then the NLRP3 protein was assessed using western blot analysis.

**Figure:**
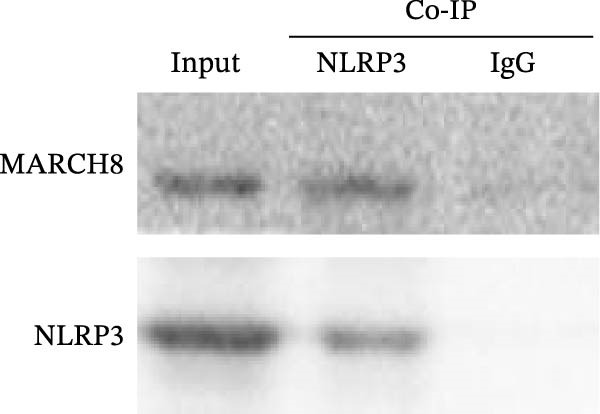
(A)

**Figure:**
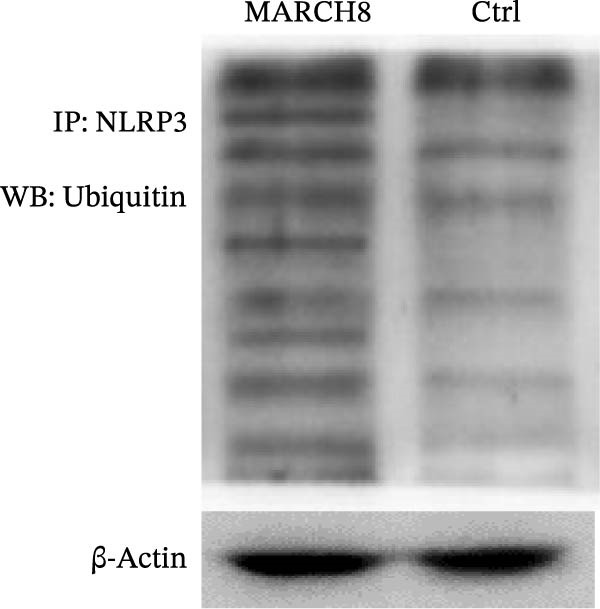
(B)

**Figure:**
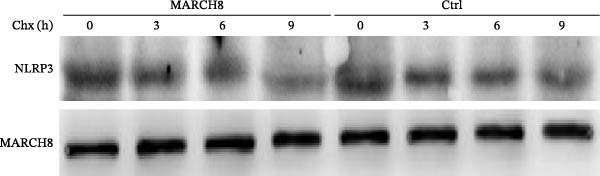
(C)

**Figure:**
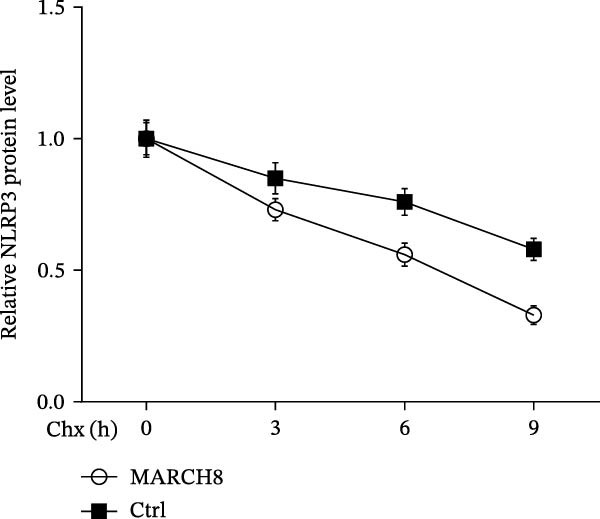
(D)

**Figure:**
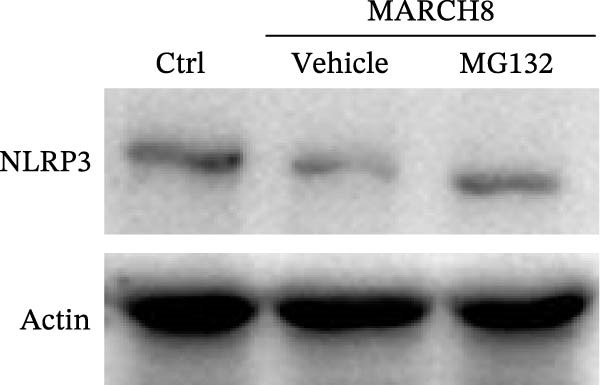
(E)

## 4. Discussion

In the pathogenesis of atherosclerosis, both oxidative stress and inflammation play important roles in plaque development, contributing to endothelial dysfunction and macrophage recruitment [38]. Emerging studies have demonstrated that several medicinal plants and their active components contribute to alleviating atherosclerosis. Qixian granule mitigates postmenopausal atherosclerosis by inhibiting GSH peroxidase 4 and ferritin heavy chain‐dependent ferroptosis in endothelial cells [39]. Orientin decreases ox‐LDL–triggered endothelial cell death by activating Sestrin 1‐dependent autophagy and decreasing oxidative stress and inflammation [40]. Given the regulatory role of Orientin in NLRP3 [41] and the role of the NLRP3 inflammasome in atherosclerosis [42], we investigated whether Orientin alleviates atherosclerosis through the NLRP3‐dependent pyroptosis pathway. In the current study, we demonstrated that (1) Orientin alleviated atherosclerosis and hypercholesterolemia in diabetic mice, (2) Orientin decreased HAEC apoptosis and inflammation following ox‐LDL/HG challenge, (3) Orientin protected against ox‐LDL/HG–induced oxidative stress and EndMT, (4) Orientin attenuated ROS‐triggered pyroptosis in HAECs, (5) Orientin inhibited NLRP3 inflammasome activation, and (6) MARCH8 promoted ubiquitination and proteasomal degradation of NLRP3 protein.

Oxidative stress is an imbalance of ROS generation and elimination and acts as a key risk factor for the development of atherosclerosis [43]. ROS trigger several types of cell death such as apoptosis, ferroptosis, and pyroptosis, all of which are implicated in the progression of atherosclerosis [44]. Specifically, endothelial apoptosis triggers plaque erosion and precipitates thrombotic events [45, 46]. Pyroptosis, on the other hand, activates endothelial cells, leading to the recruitment of monocytes and inflammatory cells [47]. Furthermore, HG‐induced necroptosis of endothelial cells increases endothelium permeability [32, 48]. Orientin has been demonstrated to regulate apoptosis [49]. However, whether Orientin regulates cell death in the atherosclerosis process is rarely reported. In the study, we found that Orientin plays a regulatory role in NLRP3‐mediated pyroptosis. This not only demonstrates the function of Orientin in regulating cell death other than apoptosis but also uncovers the potential of Orientin to modulate cell death in atherosclerosis.

The role of medicinal plant‐derived natural products in regulating ROS is multifaceted. On the one hand, certain natural products, such as flavonoids, have been shown to scavenge ROS in the digestive tract [50]. Conversely, compounds like corynoline and cinnamaldehyde have been utilized to inhibit cancer cell growth due to their ability to induce ROS generation [51, 52]. This discrepancy raises an intriguing question: Given that most natural products exhibit antioxidant and anti‐inflammatory properties, why do some promote ROS generation? The answer may lie in the interactions these products have with different cellular components, which deserve to be further explored. Nowadays, the majority of natural products are examined for their antioxidant properties in atherosclerosis, with a focus on their ability to reduce ROS levels [1]. For instance, baicalin has been shown to mitigate atherosclerosis by inhibiting ROS production and subsequent NLRP3‐mediated pyroptosis [53]. Similarly, paeonol reduces the production of the microbial metabolite α‐hydroxyisobutyric acid, thereby alleviating endothelial inflammation in atherosclerosis mediated by the ROS/thioredoxin‐interacting protein (TXNIP)/NLRP3 pathway [54]. The paradoxical role of natural products in ROS regulation raises significant concerns about their use in atherosclerosis. Orientin has been previously reported to reduce ROS levels in osteoclasts [55] and mouse pheochromocytoma cell line cells [49]. However, no research has been conducted to date on how Orientin regulates ROS in atherosclerosis. In the study, we demonstrated that Orientin suppresses ROS generation and the resultant pyroptosis. Furthermore, Orientin ameliorates atherosclerosis and hypercholesterolemia in diabetic mice, as evidenced by decreases in total cholesterol, LDL‐cholesterol, and triglyceride levels, and an increase in blood levels of high‐density lipoprotein (data not shown).

The nontranscriptional priming and activation of the NLRP3 inflammasome are increasingly recognized to be partially regulated by PTMs, including sumoylation, phosphorylation, and ubiquitylation [56]. Research has shown that E3 ligases such as F‐box/LRR‐repeat protein 2 (FBXL2) [57] and tripartite motif‐containing protein 31 (TRIM31) [58] interact with NLRP3, leading to subsequent ubiquitylation and proteasomal degradation. In the study, we have demonstrated that Orientin regulates the NLRP3 inflammasome via MARCH8, thereby providing a novel pathway through which Orientin modulates NLRP3. The limitations of our study can be summarized as follows: (1) Orientin may play a role in other types of cell death induced by ROS, (2) the underlying mechanism of Orientin in regulating EndMT remains unknown, and (3) it remains unclear to what extent studies using HAECs can explain their roles in diabetic atherosclerosis in vivo. Therefore, it is necessary to ascertain the function of Orientin on various types of diabetic animal models. Moreover, based on the pharmacokinetic data obtained following intraperitoneal administration of Orientin, it is essential to ascertain the optimal concentration for this administration route. Future research should address these limitations and aim to elucidate the regulatory networks of Orientin in alleviating atherosclerosis.

## Author Contributions

Qi Li, Lei Zhang, Fu‐Chen Song, and Han‐Zhi Lu contributed to the study conception and design. Material preparation, data collection, and analysis were performed by Qi Li and Min Gao. The first draft of the manuscript was written by Qi Li, Lei Zhang, and Han‐Zhi Lu.

## Funding

This work was supported by the National Science and Technology Major Project “Research on modernization of traditional Chinese Medicine” (Grant 2019YFC1711604), the Clinical Science and Technology Innovation Program of Shanghai Shen Kang Hospital Development Center (Grant SHDC12019X33), and the Advantageous Specialty Construction Project of TCM‐Vascular Surgery of Yueyang Hospital of Integrated Traditional Chinese and Western Medicine (Grant YW211.03.06).

## Disclosure

All authors read and approved the final manuscript.

## Ethics Statement

The study on rats was approved by the ethical committee of Shanghai University of Traditional Chinese Medicine (Number YYLAC‐2023‐355‐1) in accordance with the ARRIVE guidelines to minimize animal suffering.

## Conflicts of Interest

The authors declare no conflicts of interest.

## Supporting Information

Additional supporting information can be found online in the Supporting Information section.

## Supporting information


**Supporting Information 1** Table S1: The qRT‐PCR primers used in the study.

**Supporting Information 2** Table S2: Forty‐one E3 ubiquitin ligases targeting the NLRP3 protein were predicted by the UbiBrowser tool (http://ubibrowser.bio-it.cn/ubibrowser/).

**Supporting Information 3** Table S3: Differentially expressed genes were identified in oxLDL/HG‐treated endothelial cells using a publicly available dataset (GSE173669).

**Supporting Information 4** Figure S1: Orientin treatment increased MARCH8 expression. (A) Venn diagram analysis identified that three E3 ubiquitin ligases (CBLC, CRYAB, and MARCH8) were dysregulated in the GSE173669 dataset. (B and C) Orientin treatment increased MARCH8 expression at the mRNA levels (B) and protein levels (C) but did not affect CBLC and CRYAB expression.

**Supporting Information 5** Figure S2: MARCH8 knockdown decreased NLRP3 expression. (A) MARCH8 overexpression increased NLRP3 mRNA expression. (B and C) MARCH8 knockdown downregulated NLRP3 protein expression.

## Data Availability

All relevant data supporting the conclusions of this article are included within the manuscript.

## References

[1] Zhang Q. , Liu J. , Duan H. , Li R. , Peng W. , and Wu C. , Activation of Nrf2/HO-1 Signaling: An Important Molecular Mechanism of Herbal Medicine in the Treatment of Atherosclerosis via the Protection of Vascular Endothelial Cells From Oxidative Stress, Journal of Advanced Research. (2021) 34, 43–63, 10.1016/j.jare.2021.06.023. 35024180 PMC8655139

[2] Jebari-Benslaiman S. , Galicia-Garcia U. , and Larrea-Sebal A. , et al.Pathophysiology of Atherosclerosis, International Journal of Molecular Sciences. (2022) 23, no. 6, 10.3390/ijms23063346, 3346. 35328769 PMC8954705

[3] Bergheanu S. C. , Bodde M. C. , and Jukema J. W. , Pathophysiology and Treatment of Atherosclerosis: Current View and Future Perspective on Lipoprotein Modification Treatment, Netherlands Heart Journal. (2017) 25, no. 4, 231–242. 28194698 10.1007/s12471-017-0959-2 PMC5355390

[4] Poznyak A. V. , Bharadwaj D. , Prasad G. , Grechko A. V. , Sazonova M. A. , and Orekhov A. N. , Renin-Angiotensin System in Pathogenesis of Atherosclerosis and Treatment of CVD, International Journal of Molecular Sciences. (2021) 22, no. 13, 10.3390/ijms22136702, 6702. 34206708 PMC8269397

[5] Wong J. F. and Simmons C. A. , Microfluidic Assay for the on-Chip Electrochemical Measurement of Cell Monolayer Permeability, Lab on a Chip. (2019) 19, no. 6, 1060–1070, 10.1039/C8LC01321G, 2-s2.0-85062859580. 30778462

[6] Lorey M. B. , Öörni K. , and Kovanen P. T. , Modified Lipoproteins Induce Arterial Wall Inflammation During Atherogenesis, Frontiers in Cardiovascular Medicine. (2022) 9, 10.3389/fcvm.2022.841545, 841545. 35310965 PMC8927694

[7] Chang X. , Zhang T. , Zhang W. , Zhao Z. , and Sun J. , Natural Drugs as a Treatment Strategy for Cardiovascular Disease Through the Regulation of Oxidative Stress, Oxidative Medicine and Cellular Longevity. (2020) 2020, 10.1155/2020/5430407, 5430407. 33062142 PMC7537704

[8] Kerry N. L. and Abbey M. , Red Wine and Fractionated Phenolic Compounds Prepared From Red Wine Inhibit Low Density Lipoprotein Oxidation In Vitro, Atherosclerosis. (1997) 135, no. 1, 93–102, 10.1016/S0021-9150(97)00156-1, 2-s2.0-0030731353. 9395277

[9] Lam K. Y. , Ling A. P. K. , Koh R. Y. , Wong Y. P. , and Say Y. H. , A Review on Medicinal Properties of Orientin, Advances in Pharmacological Sciences. (2016) 2016, 10.1155/2016/4104595, 2-s2.0-84973537481, 4104595. 27298620 PMC4889806

[10] Li C. , Cai C. , Zheng X. , Sun J. , and Ye L. , Orientin Suppresses Oxidized Low-Density Lipoproteins Induced Inflammation and Oxidative Stress of Macrophages in Atherosclerosis, Bioscience, Biotechnology, and Biochemistry. (2020) 84, no. 4, 774–779, 10.1080/09168451.2019.1702871. 31829093

[11] Ku S.-K. , Kwak S. , and Bae J.-S. , Orientin Inhibits High Glucose-Induced Vascular Inflammation In Vitro and In Vivo, Inflammation. (2014) 37, no. 6, 2164–2173, 10.1007/s10753-014-9950-x, 2-s2.0-84937549362. 24950780

[12] Lee W. , Ku S.-K. , and Bae J.-S. , Vascular Barrier Protective Effects of Orientin and Isoorientin in LPS-Induced Inflammation In Vitro and In Vivo, Vascular Pharmacology. (2014) 62, no. 1, 3–14, 10.1016/j.vph.2014.04.006, 2-s2.0-84902124357. 24792192

[13] Fu J. and Wu H. , Structural Mechanisms of NLRP3 Inflammasome Assembly and Activation, Annual Review of Immunology. (2023) 41, no. 1, 301–316, 10.1146/annurev-immunol-081022-021207. PMC10159982 36750315

[14] Kelley N. , Jeltema D. , Duan Y. , and He Y. , The NLRP3 Inflammasome: An Overview of Mechanisms of Activation and Regulation, International Journal of Molecular Sciences. (2019) 20, no. 13, 10.3390/ijms20133328, 2-s2.0-85069291313, 3328. 31284572 PMC6651423

[15] Neudorf H. and Little J. P. , Impact of Fasting & Ketogenic Interventions on the NLRP3 Inflammasome: A Narrative Review, Biomedical Journal. (2024) 47, no. 1, 10.1016/j.bj.2023.100677, 100677. 37940045 PMC10821592

[16] Xu X. , Yang Y. , and Wang G. , et al.Low Shear Stress Regulates Vascular Endothelial Cell Pyroptosis Through miR-181b-5p/STAT-3 Axis, Journal of Cellular Physiology. (2021) 236, no. 1, 318–327, 10.1002/jcp.29844. 32510626

[17] Tang J. , Li T. , and Xiong X. , et al.Colchicine Delivered by a Novel Nanoparticle Platform Alleviates Atherosclerosis by Targeted Inhibition of NF-KappaB/NLRP3 Pathways in Inflammatory Endothelial Cells, Journal of Nanobiotechnology. (2023) 21, no. 1, 10.1186/s12951-023-02228-z, 460. 38037046 PMC10690998

[18] Wang L. , Zhao X. , and Ding J. , et al.Oridonin Attenuates the Progression of Atherosclerosis by Inhibiting NLRP3 and Activating Nrf2 in Apolipoprotein E-Deficient Mice, Inflammopharmacology. (2023) 31, no. 4, 1993–2005, 10.1007/s10787-023-01161-9. 37155118 PMC10352416

[19] Zhang Y. , Zhu Z. , and Cao Y. , et al.Rnd3 Suppresses Endothelial Cell Pyroptosis in Atherosclerosis Through Regulation of Ubiquitination of TRAF6, Clinical and Translational Medicine. (2023) 13, no. 9, 10.1002/ctm2.1406, e1406. 37743632 PMC10518494

[20] Xiao Q. , Cui Y. , Zhao Y. , Liu L. , Wang H. , and Yang L. , Orientin Relieves Lipopolysaccharide-Induced Acute Lung Injury in Mice: The Involvement of Its Anti-Inflammatory and Anti-Oxidant Properties, International Immunopharmacology. (2021) 90, 10.1016/j.intimp.2020.107189, 107189. 33214095

[21] Wang Z.-C. , Niu K.-M. , and Wu Y.-J. , et al.A Dual Keap1 and p47(phox) Inhibitor Ginsenoside Rb1 Ameliorates High Glucose/Ox-LDL-Induced Endothelial Cell Injury and Atherosclerosis, Cell Death & Disease. (2022) 13, no. 9, 10.1038/s41419-022-05274-x, 824. 36163178 PMC9512801

[22] Livak K. J. and Schmittgen T. D. , Analysis of Relative Gene Expression Data Using Real-Time Quantitative PCR and the 2^−ΔΔC^ _T_ Method, Methods. (2001) 25, no. 4, 402–408, 10.1006/meth.2001.1262, 2-s2.0-0035710746. 11846609

[23] Song F.-C. , Yuan J.-Q. , and Zhu M.-D. , et al.High Glucose Represses the Proliferation of Tendon Fibroblasts by Inhibiting Autophagy Activation in Tendon Injury, Bioscience Reports. (2022) 42, no. 3, 10.1042/BSR20210640. PMC8935382 35293974

[24] Sitia S. , Tomasoni L. , and Atzeni F. , et al.From Endothelial Dysfunction to Atherosclerosis, Autoimmunity Reviews. (2010) 9, no. 12, 830–834, 10.1016/j.autrev.2010.07.016, 2-s2.0-77956934512. 20678595

[25] Mudau M. , Genis A. , Lochner A. , and Strijdom H. , Endothelial Dysfunction: The Early Predictor of Atherosclerosis, Cardiovascular Journal of Africa. (2012) 23, no. 4, 222–231, 10.5830/CVJA-2011-068, 2-s2.0-84861323605. 22614668 PMC3721957

[26] Higashi Y. , Roles of Oxidative Stress and Inflammation in Vascular Endothelial Dysfunction-Related Disease, Antioxidants. (2022) 11, no. 10, 10.3390/antiox11101958, 1958. 36290681 PMC9598825

[27] Shaito A. , Aramouni K. , and Assaf R. , et al.Oxidative Stress-Induced Endothelial Dysfunction in Cardiovascular Diseases, Frontiers in Bioscience-Landmark. (2022) 27, no. 3, 10.31083/j.fbl2703105, 105. 35345337

[28] Liu H.-T. , Zhou Z.-X. , and Ren Z. , et al.EndMT: Potential Target of H_2_S Against Atherosclerosis, Current Medicinal Chemistry. (2021) 28, no. 18, 3666–3680, 10.2174/0929867327999201116194634. 33200693

[29] Souilhol C. , Harmsen M. C. , Evans P. C. , and Krenning G. , Endothelial–Mesenchymal Transition in Atherosclerosis, Cardiovascular Research. (2018) 114, no. 4, 565–577, 10.1093/cvr/cvx253, 2-s2.0-85042944375. 29309526

[30] Gong L. , Lei Y. , and Liu Y. , et al.Vaccarin Prevents Ox-LDL-Induced HUVEC EndMT, Inflammation and Apoptosis by Suppressing ROS/p38 MAPK Signaling, American Journal of Translational Research. (2019) 11, no. 4, 2140–2154. 31105824 PMC6511755

[31] Zheng D. , Liu J. , Piao H. , Zhu Z. , Wei R. , and Liu K. , ROS-Triggered Endothelial Cell Death Mechanisms: Focus on Pyroptosis, Parthanatos, and Ferroptosis, Frontiers in Immunology. (2022) 13, 10.3389/fimmu.2022.1039241, 1039241. 36389728 PMC9663996

[32] Lin J. , Chen M. , and Liu D. , et al.Exogenous Hydrogen Sulfide Protects Human Umbilical Vein Endothelial Cells Against High Glucose-Induced Injury by Inhibiting the Necroptosis Pathway, International Journal of Molecular Medicine. (2018) 41, no. 3, 1477–1486, 10.3892/ijmm.2017.3330, 2-s2.0-85041004449. 29286079 PMC5819925

[33] Wu X. , Zhang H. , and Qi W. , et al.Nicotine Promotes Atherosclerosis via ROS-NLRP3-Mediated Endothelial Cell Pyroptosis, Cell Death & Disease. (2018) 9, no. 2, 10.1038/s41419-017-0257-3, 2-s2.0-85041693664, 171. 29416034 PMC5833729

[34] Fang S. , Wan X. , and Zou X. , et al.Arsenic Trioxide Induces Macrophage Autophagy and Atheroprotection by Regulating ROS-Dependent TFEB Nuclear Translocation and AKT/mTOR Pathway, Cell Death & Disease. (2021) 12, no. 1, 10.1038/s41419-020-03357-1, 88. 33462182 PMC7814005

[35] Zhang L. , Ko C.-J. , and Li Y. , et al.Peli1 Facilitates NLRP3 Inflammasome Activation by Mediating ASC Ubiquitination, Cell Reports. (2021) 37, no. 4, 10.1016/j.celrep.2021.109904, 109904. 34706239 PMC12011377

[36] Lamkanfi M. and Dixit V. M. , Mechanisms and Functions of Inflammasomes, Cell. (2014) 157, no. 5, 1013–1022, 10.1016/j.cell.2014.04.007, 2-s2.0-84901310586. 24855941

[37] Bednash J. S. and Mallampalli R. K. , Regulation of Inflammasomes by Ubiquitination, Cellular & Molecular Immunology. (2016) 13, no. 6, 722–728, 10.1038/cmi.2016.15, 2-s2.0-84994620550. 27063466 PMC5101450

[38] Mury P. , Chirico E. N. , Mura M. , Millon A. , Canet-Soulas E. , and Pialoux V. , Oxidative Stress and Inflammation, Key Targets of Atherosclerotic Plaque Progression and Vulnerability: Potential Impact of Physical Activity, Sports Medicine. (2018) 48, no. 12, 2725–2741, 10.1007/s40279-018-0996-z, 2-s2.0-85055314541. 30302720

[39] Zhang M. , Mao C. , Dai Y. , Xu X. , and Wang X. , Qixian Granule Inhibits Ferroptosis in Vascular Endothelial Cells by Modulating TRPML1 in the Lysosome to Prevent Postmenopausal Atherosclerosis, Journal of Ethnopharmacology. (2024) 328, 118076. 38521431 10.1016/j.jep.2024.118076

[40] Gao F. , Zhao Y. , and Zhang B. , et al.Orientin Alleviates Ox-LDL-Induced Oxidative Stress, Inflammation and Apoptosis in Human Vascular Endothelial Cells by Regulating Sestrin 1 (SESN1)-Mediated Autophagy, Journal of Molecular Histology. (2024) 55, no. 1, 109–120, 10.1007/s10735-023-10176-z. 38165567

[41] Xiao Q. , Qu Z. , Zhao Y. , Yang L. , and Gao P. , Orientin Ameliorates LPS-Induced Inflammatory Responses Through the Inhibitory of the NF-κB Pathway and NLRP3 Inflammasome, Evidence-Based Complementary and Alternative Medicine. (2017) 2017, no. 1, 10.1155/2017/2495496, 2-s2.0-85011610036, 2495496. 28197210 PMC5288532

[42] Grebe A. , Hoss F. , and Latz E. , NLRP3 Inflammasome and the IL-1 Pathway in Atherosclerosis, Circulation Research. (2018) 122, no. 12, 1722–1740, 10.1161/CIRCRESAHA.118.311362, 2-s2.0-85051799689. 29880500

[43] Batty M. , Bennett M. R. , and Yu E. , The Role of Oxidative Stress in Atherosclerosis, Cells. (2022) 11, no. 23, 10.3390/cells11233843, 3843. 36497101 PMC9735601

[44] Li M. , Wang Z.-W. , Fang L.-J. , Cheng S.-Q. , Wang X. , and Liu N.-F. , Programmed Cell Death in Atherosclerosis and Vascular Calcification, Cell Death & Disease. (2022) 13, no. 5, 10.1038/s41419-022-04923-5, 467. 35585052 PMC9117271

[45] Irani K. , Oxidant Signaling in Vascular Cell Growth, Death, and Survival: A Review of the Roles of Reactive Oxygen Species in Smooth Muscle and Endothelial Cell Mitogenic and Apoptotic Signaling, Circulation Research. (2000) 87, no. 3, 179–183, 10.1161/01.RES.87.3.179, 2-s2.0-0033886373. 10926866

[46] Bombeli T. , Karsan A. , Tait J. F. , and Harlan J. M. , Apoptotic Vascular Endothelial Cells Become Procoagulant, Blood. (1997) 89, no. 7, 2429–2442, 10.1182/blood.V89.7.2429. 9116287

[47] Yin Y. , Li X. , and Sha X. , et al.Early Hyperlipidemia Promotes Endothelial Activation via a Caspase-1-Sirtuin 1 Pathway, Arteriosclerosis, Thrombosis, and Vascular Biology. (2015) 35, no. 4, 804–816, 10.1161/ATVBAHA.115.305282, 2-s2.0-84933513721. 25705917 PMC4376583

[48] Xu Y.-J. , Zheng L. , Hu Y.-W. , and Wang Q. , Pyroptosis and Its Relationship to Atherosclerosis, Clinica Chimica Acta. (2018) 476, 28–37, 10.1016/j.cca.2017.11.005, 2-s2.0-85034016224. 29129476

[49] Qi S. , Feng Z. , Li Q. , Qi Z. , and Zhang Y. , Inhibition of ROS-Mediated Activation Src-MAPK/AKT Signaling by Orientin Alleviates H_2_O_2_-Induced Apoptosis in PC12 cells, Drug Design, Development and Therapy. (2018) 12, 3973–3984, 10.2147/DDDT.S178217, 2-s2.0-85057847021. 30510405 PMC6248275

[50] Pietta P.-G. , Flavonoids as Antioxidants, Journal of Natural Products. (2000) 63, no. 7, 1035–1042, 10.1021/np9904509, 2-s2.0-0033859612. 10924197

[51] Yi C. , Li X. , Chen S. , Liu M. , Lu W. , and Ye X. , Natural Product Corynoline Suppresses Melanoma Cell Growth Through Inducing Oxidative Stress, Phytotherapy Research. (2020) 34, no. 10, 2766–2777, 10.1002/ptr.6719. 32430958

[52] Ka H. , Park H.-J. , and Jung H.-J. , et al.Cinnamaldehyde Induces Apoptosis by ROS-Mediated Mitochondrial Permeability Transition in Human Promyelocytic Leukemia HL-60 Cells, Cancer Letters. (2003) 196, no. 2, 143–152, 10.1016/S0304-3835(03)00238-6, 2-s2.0-0037969283. 12860272

[53] Zhao J. , Wang Z. , Yuan Z. , Lv S. , and Su Q. , Baicalin Ameliorates Atherosclerosis by Inhibiting NLRP3 Inflammasome in Apolipoprotein E-Deficient Mice, Diabetes and Vascular Disease Research. (2020) 17, no. 6, 10.1177/1479164120977441, 1479164120977441. 33269624 PMC7919226

[54] Liu Y. , Wu H. , and Wang T. , et al.Paeonol Reduces Microbial Metabolite α-Hydroxyisobutyric Acid to Alleviate the ROS/TXNIP/NLRP3 Pathway-Mediated Endothelial Inflammation in Atherosclerosis Mice, Chinese Journal of Natural Medicines. (2023) 21, no. 10, 759–774, 10.1016/S1875-5364(23)60506-0. 37879794

[55] Zheng Y. , Wang X. , Pan Y. J. , Shi X. F. , Yang L. , and Lou Y. L. , Orientin Suppresses Osteoclastogenesis and Ameliorates Ovariectomy-Induced Osteoporosis via Suppressing ROS Production, Food Science & Nutrition. (2023) 11, no. 9, 5582–5595, 10.1002/fsn3.3516. 37701239 PMC10494641

[56] Swanson K. V. , Deng M. , and Ting J. P.-Y. , The NLRP3 Inflammasome: Molecular Activation and Regulation to Therapeutics, Nature Reviews Immunology. (2019) 19, no. 8, 477–489, 10.1038/s41577-019-0165-0, 2-s2.0-85065188040. PMC7807242 31036962

[57] Han S.H. , Lear T. B. , and Jerome J. A. , et al.Lipopolysaccharide Primes the NALP3 Inflammasome by Inhibiting Its Ubiquitination and Degradation Mediated by the SCFFBXL2 E3 Ligase, Journal of Biological Chemistry. (2015) 290, no. 29, 18124–18133, 10.1074/jbc.M115.645549, 2-s2.0-84937468257. 26037928 PMC4505057

[58] Song H. , Liu B. , and Huai W. , et al.The E3 Ubiquitin Ligase TRIM31 Attenuates NLRP3 Inflammasome Activation by Promoting Proteasomal Degradation of NLRP3, Nature Communications. (2016) 7, no. 1, 10.1038/ncomms13727, 2-s2.0-85006056503, 13727. PMC5155141 27929086

