# Supplementary material for: Orientin Mitigates High Glucose/Ox‐LDL–Triggered Endothelial Cell Injury and Atherosclerosis by Regulating MARCH8‐Mediated NLRP3 Inflammasome Activation
Source: Mediators Inflamm. 2026 Mar 27;2026:1841497. doi: 10.1155/mi/1841497 (PMC13140237; doi:10.1155/mi/1841497)
Supplement: Supplementary file 1 — Supporting Information 1 Table S1: The qRT‐PCR primers used in the study. [file MI-2026-1841497-s001.docx]

**Supporting Table S1. The qRT-PCR primers used in the study.**

| Gene symbol | Sense, 5’-3’ | Anti-sense, 5’-3’ |
| --- | --- | --- |
| IL-6 | CACTGGTCTTTTGGAGTTTGAGG | AGGCTGGCATTTGTGGTTG |
| TNF-α | GCTGGAGAAGGGTGACCGAC | CAGGGCAATGATCCCAAAGTA |
| ICAM-1 | CTCTCCCCCCGGTATGAGATT | GTGGCTTGTGTGTTCGGTTTC |
| VCAM-1 | AAGAAAAAAGCGGAGACAGGAG | AGGATGCAAAATAGAGCACGAG |
| CD31 | ACCAAGATAGCCTCAAAGTCGG | CTGGGAGAGCATTTCACATACG |
| vWF | ACTTTGAGGTGGTGGAGTCTGG | TGCTGGTGAGGTCATTGTTCTG |
| α-sma | CAGGGGGTGATGGTGGGAAT | GTGAGCAGGGTGGGATGCT |
| vimentin | CTGGATTCACTCCCTCTGGTTG | CGTGATGCTGAGAAGTTTCGTTG |
| IL-18 | AGGTATGGCTGTAACTATCTCTGTG | TTGCATCTTATTATCATGTCCTGG |
| IL-1β | AGTGGTGTTCTCCATGTCCTTTC | ATCGCTTTTCCATCTTCTTCTTT |
| NLRP3 | AGGAAGATGATGTTGGACTGGG | GGAAGAGGATTCTGGAGGGTTT |
| ASC | CCACCAACCCAAGCAAGATG | TCCGCTCCAGGTCCTCCAC |
| March8 | TAAGACCAAAGAAAAGGAGAGGG | CGTGATAGAAGTGCGAGAGAAGG |
| β-actin | CCGTTGCCCTGAGGCTCTTTT | TGCGGATGTCCACGTCACACT |
