# Supplementary material for: Orientin Mitigates High Glucose/Ox‐LDL–Triggered Endothelial Cell Injury and Atherosclerosis by Regulating MARCH8‐Mediated NLRP3 Inflammasome Activation
Source: Mediators Inflamm. 2026 Mar 27;2026:1841497. doi: 10.1155/mi/1841497 (PMC13140237; doi:10.1155/mi/1841497)
Supplement: Supplementary file 4 — Supporting Information 4 Figure S1: Orientin treatment increased MARCH8 expression. (A) Venn diagram analysis identified that three E3 ubiquitin ligases (CBLC, CRYAB, and MARCH8) were dysregulated in the GSE173669 dataset. (B and C) Orientin treatment increased MARCH8 expression at the mRNA levels (B) and protein levels (C) but did not affect CBLC and CRYAB expression. [file MI-2026-1841497-s004.pptx]

## Slide 1
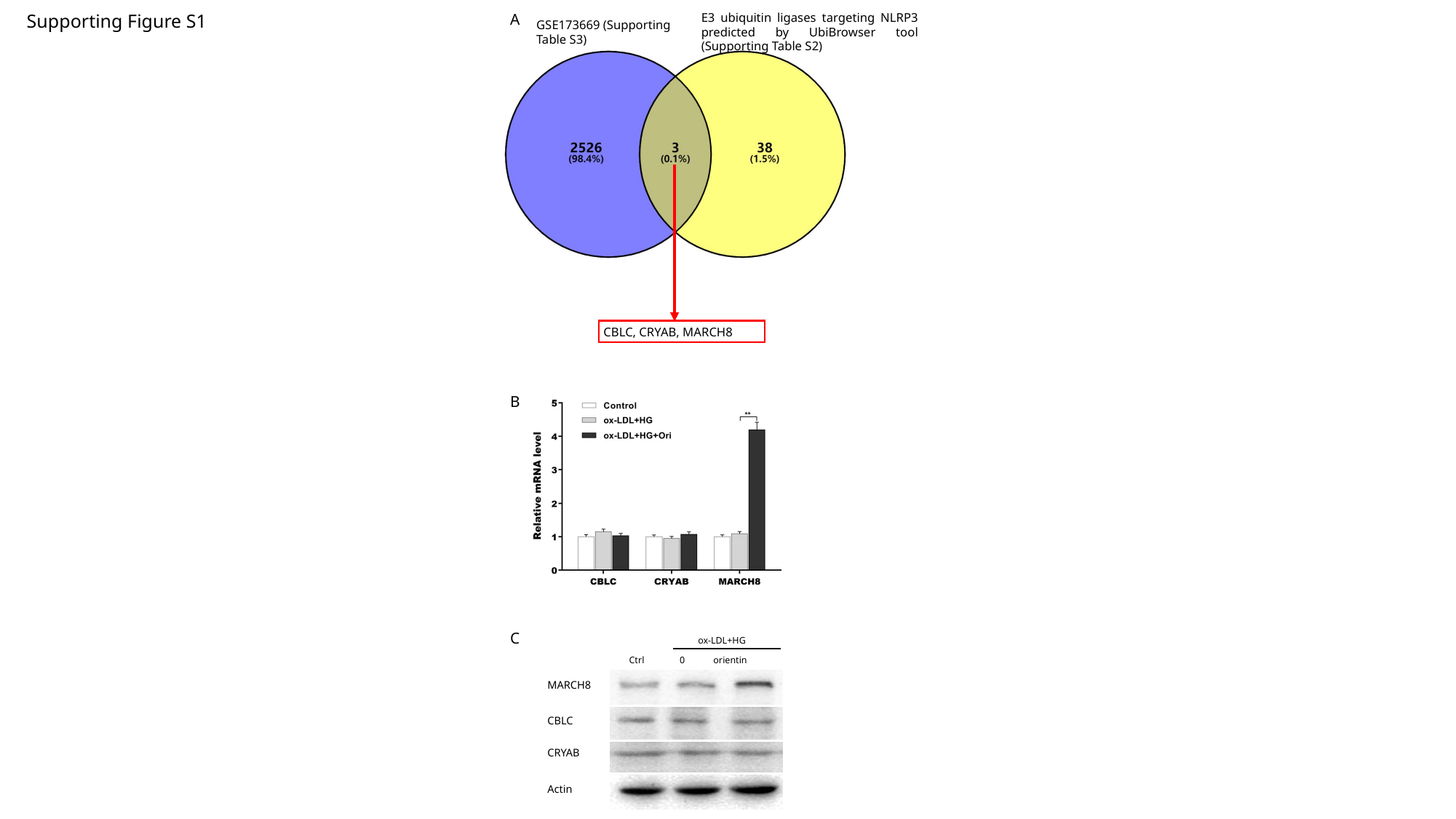

E3 ubiquitin ligases targeting NLRP3 predicted by UbiBrowser tool (Supporting Table S2)
GSE173669 (Supporting Table S3)
CBLC, CRYAB, MARCH8
Supporting Figure S1
A
B
C
 ox-LDL+HG
 Ctrl 0 orientin
MARCH8
CBLC
CRYAB
Actin
