# Supplementary figures and images for: Orientin Mitigates High Glucose/Ox‐LDL–Triggered Endothelial Cell Injury and Atherosclerosis by Regulating MARCH8‐Mediated NLRP3 Inflammasome Activation
Source: Mediators Inflamm. 2026 Mar 27;2026:1841497. doi: 10.1155/mi/1841497 (PMC13140237; doi:10.1155/mi/1841497)

## Slide 1
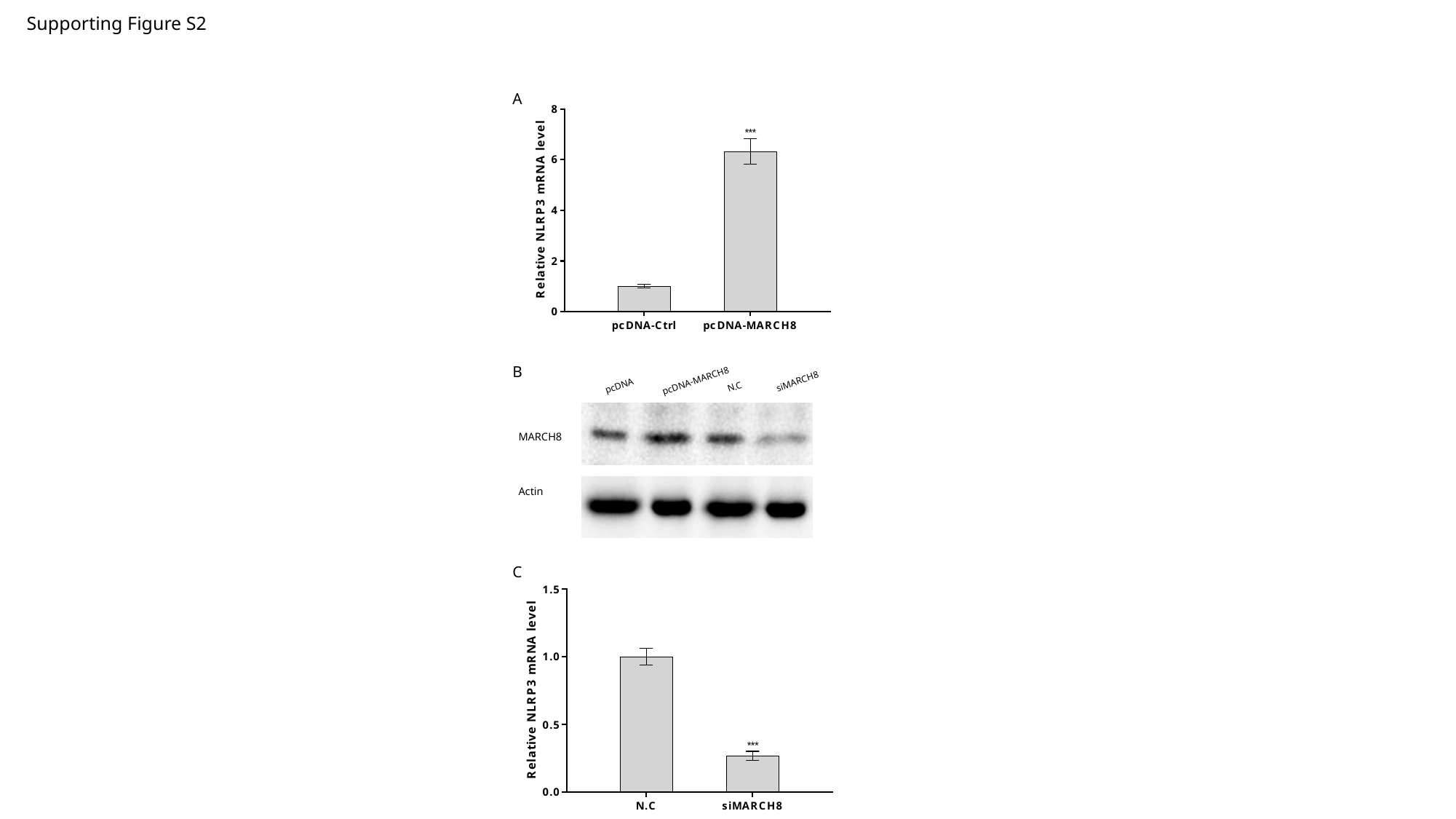

Supporting Figure S2
A
B
C
siMARCH8
N.C
pcDNA-MARCH8
 pcDNA
MARCH8
Actin

Supplement: Supplementary file 5 — Supporting Information 5 Figure S2: MARCH8 knockdown decreased NLRP3 expression. (A) MARCH8 overexpression increased NLRP3 mRNA expression. (B and C) MARCH8 knockdown downregulated NLRP3 protein expression. [file MI-2026-1841497-s003.pptx]
